# *Aebp1* loss in osteoprogenitors leads to skeletal defects resembling Ehlers-Danlos Syndrome by diminishing Wnt/**β**-catenin signaling

**DOI:** 10.1172/jci.insight.191606

**Published:** 2025-11-13

**Authors:** Shuhao Feng, Zihang Feng, Zhonghao Deng, Yiran Wei, Ru Lian, Yangchen Jin, Shiqi Zhao, Yu Jin, Zhongmin Zhang, Liang Zhao

**Affiliations:** 1 Division of Orthopaedic Surgery, Department of Orthopaedics, Nanfang Hospital, Southern Medical University, Guangzhou, Guangdong Province, China.; 2 Department of Sports Medicine, Wuhan Fourth Hospital, Wuhan, Hubei Province, China.; 3 Department of Orthopedics, The Tenth Affiliated Hospital, Southern Medical University (Dongguan People’s Hospital), Dongguan, Guangdong Province, China.; 4 Department of Orthopedics, The First Affiliated Hospital of USTC, Division of Life Sciences and Medicine, University of Science and Technology of China, Hefei, Anhui Province, China.; 5 College of pharmacy, Guangdong Pharmaceutical University, Guangzhou, Guangdong Province, China.; 6 Division of Spine Surgery, Department of Orthopaedics, Nanfang Hospital, Southern Medical University, Guangzhou, Guangdong, China.; 7 Joint Surgery Department, the First Affiliated Hospital of Guangzhou Medical University, Guangzhou, Guangdong Province, China.

**Keywords:** Bone biology, Cell biology, Development, Bone development, Osteoclast/osteoblast biology

## Abstract

Ehlers-Danlos syndrome, Classic-Like, 2 (clEDS2) is a rare genetic disorder caused by biallelic mutations in the *AEBP1* gene, which encodes aortic carboxypeptidase-like protein (ACLP). Patients with clEDS2 exhibit hallmark features such as loose connective tissues, osteoporosis, and scoliosis. Despite its clinical significance, the molecular mechanisms underlying *AEBP1* mutations in skeletal development remain poorly understood, and effective therapeutic strategies are currently unavailable. Here, using *OsxCre* conditional KO mice, we show that *Aebp1* deletion in osteoprogenitors reduces body size and bone mass, recapitulating key skeletal features reported in clEDS2. In primary osteoblasts, both genetic deletion and siRNA-mediated knockdown of *Aebp1* impair osteoblast differentiation. Mechanistically, *Aebp1* loss attenuates Wnt/β-catenin signaling in bone. Restoration of Wnt/β-catenin signaling by injecting BIO, a small molecule inhibitor of GSK3, substantially rescued bone mass reduction in *Aebp1*-KO mice. These findings support a model in which *Aebp1* sustains baseline Wnt/β-catenin tone in osteoblast-lineage cells and suggest that Wnt-targeted approaches may help mitigate clEDS2-related skeletal defects.

## Abstract 2

AEBP1 regulates bone development via Wnt/β-catenin signaling. Restoring Wnt/β-catenin signaling pathway alleviates skeletal defects in clEDS2. Offering potential therapeutic strategies for related bone disorders.

## Introduction

Ehlers-Danlos syndrome (EDS) is a heterogeneous group of inherited connective tissue disorder with considerable global morbidity, affecting approximately 1 in 5,000 individuals worldwide. However, its true prevalence may be underestimated due to frequent misdiagnosis or underdiagnosis (1–3). Clinically, EDS manifests across a broad spectrum of severity, ranging from mild symptoms to debilitating complications that profoundly impair the quality of life, including chronic pain, frequent joint dislocations, and cardiovascular abnormalities. Certain subtypes of EDS are further characterized by skeletal system involvement, such as osteoporosis and scoliosis, which exacerbate pain, elevate fracture risk, and markedly diminish patients’ overall wellbeing (4–7).

Among the diverse EDS subtypes, EDS, Classic-Like, 2 (clEDS2) represents a recently identified variant, first described by Blackburn et al. (8). Due to its rarity, epidemiological data on clEDS2 remain scarce, primarily derived from case reports and small-scale familial studies. Notably, in addition to hallmark features such as joint laxity and skin hyperextensibility, patients with clEDS2 frequently present with skeletal abnormalities, including osteoporosis and scoliosis, underscoring the subtype’s distinctive clinical profile (8, 9).

clEDS2 is an autosomal recessive condition caused by biallelic mutations in the adipocyte enhancer-binding protein 1 (*AEBP1*) gene (8, 10). The *AEBP1* gene encodes aortic carboxypeptidase-like protein (ACLP), a member of the carboxypeptidase A protein family. However, unlike other members of this family, ACLP is catalytically inactive, emphasizing its unique functional role beyond enzymatic activity (11). ACLP is known to play a critical role in extracellular matrix (ECM) organization and cellular signaling, particularly through its interaction with Wnt signaling components. Specifically, ACLP has been shown to bind the Wnt receptors FZD8 and LRP6, thereby activating canonical Wnt/β-catenin signaling (12). This interaction underscores ACLP’s pivotal role in modulating Wnt signaling, which is essential for numerous biological processes, including skeletal development. Dysfunction of ACLP due to *AEBP1* mutations results in clinical manifestations such as loose connective tissues, osteoporosis, and scoliosis, highlighting its indispensable role in maintaining skeletal structure and function (8, 10, 13). Structurally, ACLP is characterized by multiple functional domains, including a discoidin domain, which is vital for its function in ECM organization and cellular signaling (8, 11). ACLP is expressed in collagen-rich connective tissue, such as bone, the vascular system, and dermal tissues. During mouse embryonic development, ACLP has been observed in the periosteum, suggesting its potential involvement in skeletal development (14). Despite these observations, ACLP’s role in skeletal development remains unclear.

Skeletal development is a highly orchestrated and fundamental biological process that provides the structural framework necessary for support, locomotion, and protection of vital organs. This process is achieved through 2 primary mechanisms: intramembranous ossification and endochondral ossification. Intramembranous ossification governs the formation of flat bones, such as those of the skull and clavicle, where mesenchymal cells directly differentiate into osteoblasts (15–17). In contrast, endochondral ossification, which accounts for the majority of the skeleton, including long bones, involves the formation of a cartilage template that is subsequently replaced by bone (18). At the molecular level, skeletal development is regulated by an intricate network of signaling pathways, including the Hedgehog, Wnt, BMP, and FGF pathways (19). Among these, the Hedgehog signaling pathway, particularly Indian Hedgehog (Ihh), is crucial for chondrocyte proliferation and differentiation during endochondral ossification (18). Similarly, the Wnt signaling pathway plays a critical role in osteoblast differentiation and bone mass regulation, with mutations in Wnt pathway components being implicated in skeletal disorders such as osteoporosis (20). Given ACLP’s established role in activating canonical Wnt signaling, understanding its precise molecular functions in skeletal development is critical for elucidating the mechanisms underlying clEDS2-associated skeletal abnormalities and for identifying potential therapeutic targets.

Here, we investigate the role of *Aebp1* in osteoprogenitor cells and its effect on skeletal development, particularly in the context of clEDS2. Using a mouse model with targeted deletion of *Aebp1* in osteoprogenitor cells, we observed phenotypic parallels to human clEDS2, including reduced bone mass and stature. Our findings demonstrate that the loss of *Aebp1* function significantly downregulates Wnt/β-catenin signaling, contributing to the observed skeletal defects. Furthermore, we show that pharmacological activation of the Wnt/β-catenin signaling pathway suggests a potential therapeutic approach to ameliorate clEDS2-associated skeletal abnormalities.

## Results

### Loss of Aebp1 in osteoprogenitor cells results in clEDS2-related skeletal defects.

**Figure 1:**
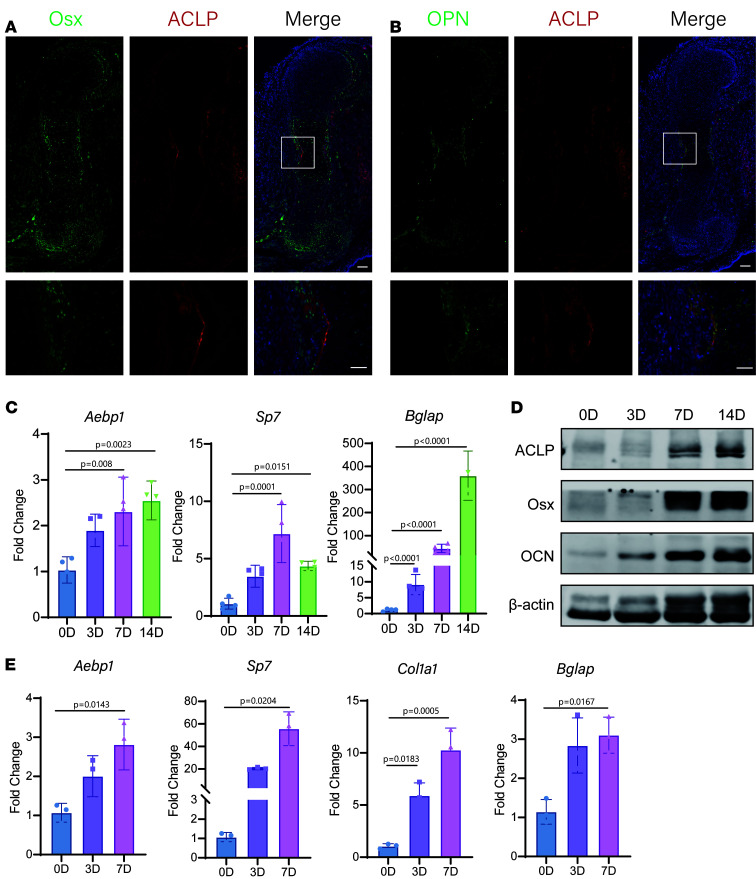
Characterization of ACLP in the developing long bones. (**A**) Representative images of Osx and ACLP IF staining of the humerus sections from E16.5 embryos with the indicated genotypes. Scale bar: 200 μm. (**B**) Representative images of OPN and ACLP IF staining of the humerus sections from E16.5 embryos with the indicated genotypes. Scale bar: 200 μm. (**C**) Relative expression of *Aebp1* and osteogenic marker genes *Sp7* and *Bglap* in mouse osteoblasts during osteogenesis were assessed by qPCR (*n* = 4, 1-way ANOVA, data shown as mean ± SD) (**D**) Western blot analysis of ACLP, Osx, and OCN protein levels during osteogenic differentiation of mouse osteoblasts. (**E**) Relative expression of *Aebp1* and osteogenic marker genes *Sp7*, *Col1a1*, and *Bglap* in MC3T3-E1 cells during osteogenesis were assessed by qPCR (*n* = 3, 1-way ANOVA, data shown as mean ± SD).

To investigate the cellular origin and molecular mechanisms underlying the skeletal phenotypes associated with clEDS2, we tested whether the bone defects could arise from loss of *Aebp1* in osteoprogenitors cells. *Aebp1* has been previously reported to be highly expressed in collagen-rich tissues (14). Our results demonstrate that ACLP expression was localized around Osterix^+^ cells (Figure 1A) and closely overlapped with osteopontin-rich (OPN-rich) bone surfaces (Figure 1B), consistent with secretion by osteoblast-lineage cells. Furthermore, during osteogenic differentiation, *Aebp1* expression increased in mouse calvaria osteoblasts (Figure 1, C and D) and in MC3T3-E1 cells (Figure 1E). These findings suggest that *Aebp1* may play a role in bone formation.

**Figure 2:**
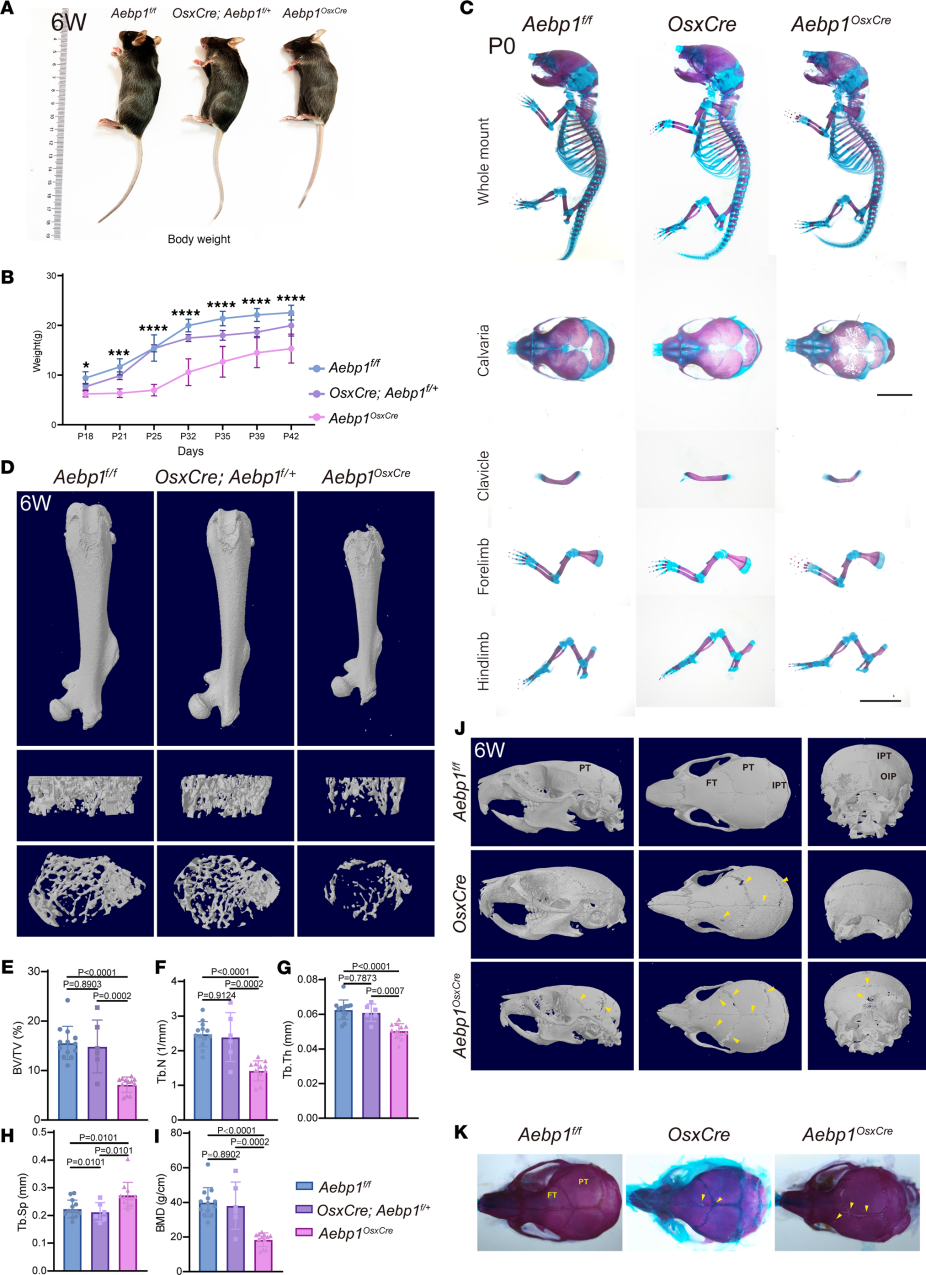
Deletion of *Aebp1* in osteoprogenitor cells induced skeletal defects. (**A**) Gross appearance of 6-week-old *Aebp1^fl/fl^* (left), *OsxCre; Aebp1^fl/+^* (middle), and *Aebp1^OsxCre^* (right) mice. (**B**) Quantification of body weight from P18 to 6-week-old mice of the indicated genotypes (*n* = 4 for each genotype, *P < 0.05, ***P < 0.001, ****P < 0.0001; 1-way ANOVA; data shown as mean ± SD). (**C**) Whole-mount Alizarin red and Alcian blue staining of *Aebp1^fl/fl^, OsxCre,* and *Aebp1^OsxCre^* mice at P0. The calvaria, clavicle, forelimb, and hindlimb were shown below. Scale bar: 1 mm. (**D**) Representative μCT images of femurs from 6-week-old littermate mice with the indicated genotypes. (**E**–**I**) Quantification of indicated parameters of μCT scanning (*n* ≥ 6 for each genotype, 1-way ANOVA, data shown as mean ± SD). (**J**) Representative μCT images of skull from 6-week-old littermate mice with the indicated genotypes. (**K**) Whole-mount Alizarin red and Alcian blue staining of littermate mice of the indicated genotypes at 6 weeks old. Yellow arrowheads indicate bone defects.

To delete *Aebp1* in osteoprogenitor cells, we utilized the *OsxCre* line (21, 22) in combination with conditional *Aebp1* (*Aebp1^fl/fl^*) mice (Supplemental Figure 1; supplemental material available online with this article; https://doi.org/10.1172/jci.insight.191606DS1). Bone tissues from *Aebp1^OsxCre^* mice showed significantly reduced ACLP expression at both the mRNA and protein levels, confirming effective recombination and knockdown (KD) (Supplemental Figure 1, C and D). The *Aebp1^OsxCre^* pups were born alive at Mendelian ratios. Postnatally, these mice were distinguishable from their WT littermates by their smaller physical size and reduced body weight. In contrast, heterozygous *OsxCre; Aebp1^fl/+^* mice appeared largely normal, with only slightly decreased body weight and shortened body length compared with WT controls (Figure 2, A and B). At P0, the *Aebp1^OsxCre^* mice displayed hypomineralized calvarial bones and clavicular hypoplasia compared with the WT and *OsxCre* littermates (Figure 2C), whereas both forelimbs and hindlimbs were comparable across groups (Figure 2C). With age, limb phenotypes of the *Aebp1^OsxCre^* mice progressed, characterized by shortened limbs (Figure 2D), reduced bone volume per tissue volume (BV/TV) (Figure 2E) and bone mineral density (BMD) (Figure 2I), lower trabecular number (Tb.N) (Figure 2F) and trabecular thickness (Tb.Th) (Figure 2G), and highertrabecular separation (Tb.Sp) (Figure 2H). Because *OsxCre; Aebp1^fl/+^* long bones were indistinguishable from WT for the analyzed μCT parameters, we used WT littermates as primary controls for long bone analyses

Next, we examined the cranial bones of adult mice. Given prior reports that *OsxCre* mice exhibit postnatal cranial bone development abnormalities (23), we first assessed *OsxCre* skulls. At P0, the cranial mineralization in *OsxCre* mice was slightly reduced compared with WT mice (Figure 2C). By 6 weeks, *OsxCre* mice displayed pronounced hypomineralization at the coronal suture, consistent with previous reports (Figure 2, J and K). However, the cranial bones of *Aebp1^OsxCre^* mice were notably smaller than both WT and *OsxCre* mice, with more pronounced hypomineralization in the coronal suture and evident hypomineralization in the occipital bone (Figure 2, J and K). Taken together, these findings demonstrate that *Aebp1* is essential in osteoprogenitor cells for normal skeletal development, and the loss of *Aebp1* function in osteoprogenitor cells may contribute to the skeletal abnormalities observed in patients with clEDS2.

### Aebp1 deletion in osteoprogenitor-lineage cells delays endochondral ossification in mice.

**Figure 3:**
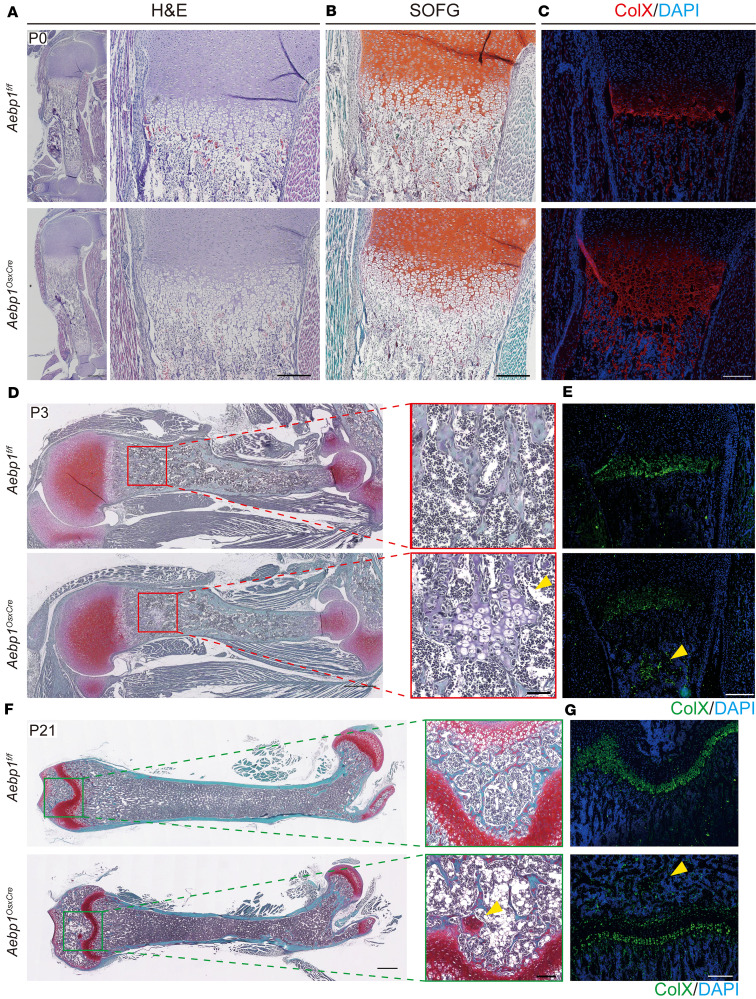
Loss of *Aebp1* in osteoprogenitor cells delayed endochondral ossification. (**A**–**C**) Representative images of H&E staining (**A**), Safranin O staining (**B**), and type X collagen IF staining (**C**) of the humerus sections from P0 pups with the indicated genotypes. Scale bar: 200 μm. (**D** and **E**) Representative images of Safranin O staining (**D**) and type X collagen IF staining (**E**) of the humerus sections from P3 pups with the indicated genotypes. Scale bar: 200 μm. (**F** and **G**) Representative images of Safranin O staining (**F**) and type X collagen IF staining (**G**) of the femur sections from P21 mice with the indicated genotypes. Scale bar: 200 μm. Arrowheads indicate regions of cartilage-like tissue.

The observation of reduced limb length and smaller body size in *Aebp1^OsxCre^* mice led us to investigate the potential alterations in endochondral ossification. Histological examination of the femur from *Aebp1^OsxCre^* mice and their littermate controls showed overall preserved tissue architecture but a notable expansion of the hypertrophic zone at P0 (Figure 3A). To interrogate chondrocyte hypertrophy in vivo, we employed Safranin O staining alongside immunofluorescence (IF) for Col10a1, a well-established marker of hypertrophic chondrocytes. The Col10a1^+^ domain, delineating hypertrophic chondrocytes, was markedly enlarged in the cartilage of *Aebp1^OsxCre^* mice at P0 (Figure 3, B and C). Because apoptosis of hypertrophic chondrocytes precedes bone formation during endochondral ossification, these data indicate a delay in this process in *Aebp1^OsxCre^* mice. Corroborating this hypothesis, Safranin O staining and IF for Col10a1 revealed aberrant cartilage-like tissue expressing Col10a1 within the bone marrow cavity of *Aebp1^OsxCre^* mice at P3, which was absent in littermate controls (Figure 3, D and E). At P21, Safranin O and Col10a1 IF staining showed persistent cartilage-like tissue in the secondary ossification center of *Aebp1^OsxCre^* mice at P21, whereas such tissue was absent in control littermates (Figure 3, F and G). In summary, *Aebp1* is required for timely endochondral ossification in osteoprogenitor-lineage cells, and its loss results in a delay consistent with the observed skeletal defects.

### Aebp1 regulates bone formation by modulating osteoblast differentiation.

**Figure 4:**
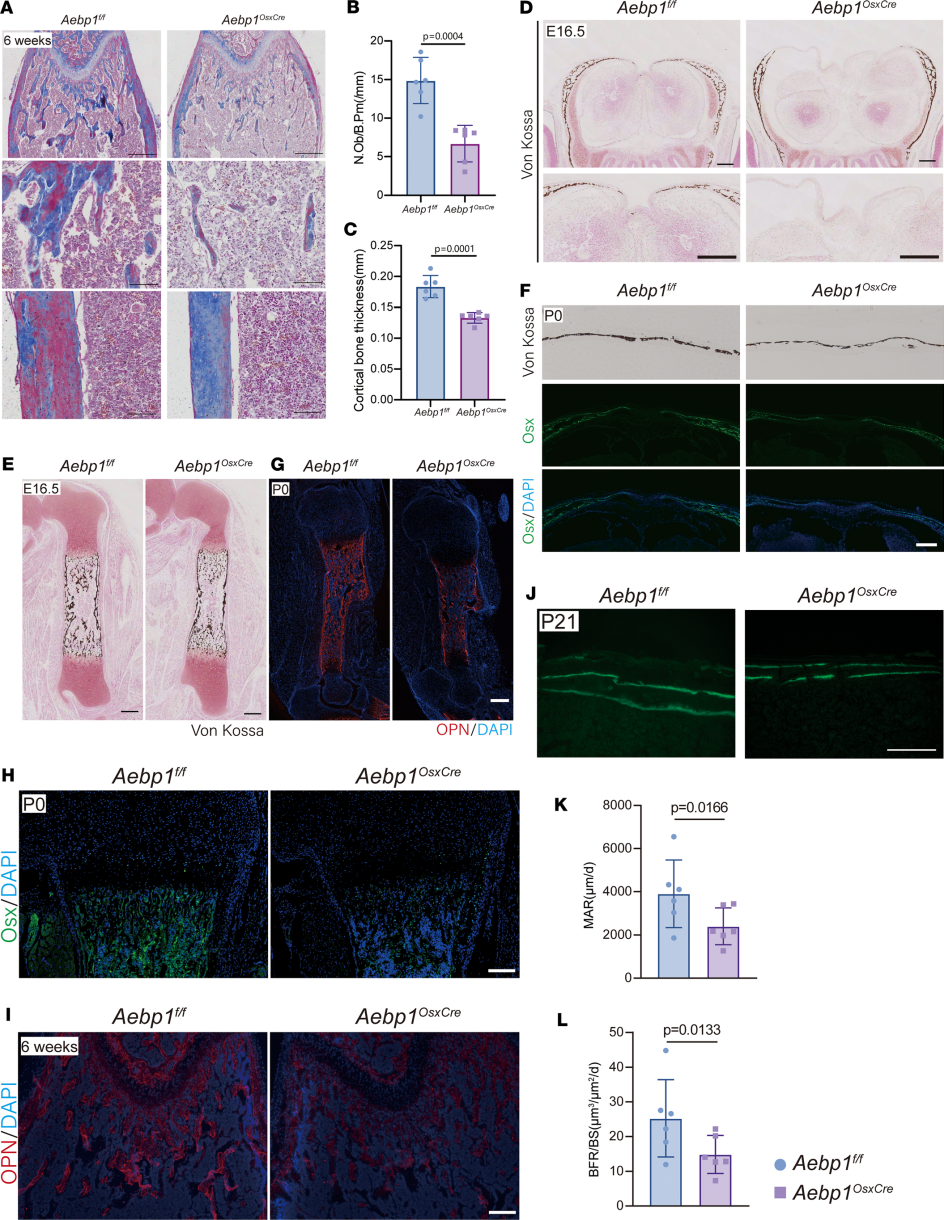
*Aebp1* is required in osteoprogenitor cells for osteoblast differentiation and bone formation. (**A**) Representative Masson’s trichrome staining of femurs from 6-week-old mice. Scale bar: 200 μm. (**B** and **C**) Quantification of N.Ob/B.Pm (**B**) and cortical bone thickness (**C**) of Masson’s trichrome staining of **A** (*n* = 6 for each genotype, Student’s *t* test, data shown as mean ± SD). (**D**) Representative images of von Kossa staining of the parietal bone sections from E16.5 embryos with the indicated genotypes. Scale bar: 200 μm. (**E**) Representative images of von Kossa staining of the femur sections from E16.5 embryos with the indicated genotypes. Scale bar: 100 μm. (**F**) Representative images of von Kossa staining (top) and Osx IF staining (bottom) of the parietal bone sections from P0 pups with the indicated genotypes. Scale bar: 200 μm. (**G**) Representative images of OPN IF staining of the humerus sections from P0 pups with the indicated genotypes. Scale bar: 100 μm. (**H**) Representative images of Osx IF staining of the humerus sections from P0 pups with the indicated genotypes. Scale bar: 200 μm. (**I**) Representative images of OPN IF staining of the femur sections from 6-week-old mice with the indicated genotypes. Scale bar: 200 μm. (**J**–**L**) Histomorphometric analysis of bone formation from 3-week-old littermate mice of indicated genotypes. Representative images of double Calcein labeling in the femur cortical bones (**J**) of indicated genotypes. Scale bar: 200 μm. Quantification of MAR (**K**) and BFR/BS (**L**) of the distal femurs from 3-week-old littermate mice of indicated genotypes (*n* = 6 for each genotype, Student’s *t* test, data shown as mean ± SD).

To elucidate whether the bone defects observed in *Aebp1^OsxCre^* mice primarily stem from impaired osteoblast differentiation, we performed in vivo analysis of osteoblast activity. Initially, osteoblast numbers were assessed using Masson’s trichrome staining. At 6 weeks of age, *Aebp1^OsxCre^* femur showed fewer osteoblasts and reduced cortical thickness versus littermate controls (Figure 4, A–C). These findings are consistent with reduced osteoblast numbers following *Aebp1* deletion. Furthermore, von Kossa staining showed reduced bone ossification in the calvaria and limb bones of *Aebp1^OsxCre^* mice at E16.5 and P0 relative to littermate controls (Figure 4, D–F). Notably, the expression of Osx (encoded by *Sp7*), a transcription factor for early osteoblast commitment, was diminished in the calvaria (Figure 4F) and long bones (Figure 4H) of *Aebp1^OsxCre^* mice at P0. Similarly, the expression of OPN was markedly reduced in the long bones of *Aebp1^OsxCre^* mice at P0 (Figure 4G) and at 6 weeks of age (Figure 4I). Bone histomorphometric analyses further substantiated these findings, revealing a pronounced reduction in calcein labeling in the bones of *Aebp1^OsxCre^* mice (Figure 4J). Both the mineral apposition rate (MAR) (Figure 4K) and the bone formation rate per bone surface (BFR/BS) (Figure 4L) were significantly diminished compared with littermate controls. Collectively, these results underscore that the skeletal defects observed in *Aebp1^OsxCre^* mice are attributable to a reduction in osteoblast numbers, coupled with impaired osteoblast differentiation and maturation.

To further test cell autonomy, we perturbed *Aebp1* in osteoblasts in vitro (Supplemental Figure 2). Initially, siRNA-mediated KD was employed to suppress *Aebp1* expression in osteoblasts, with successful downregulation confirmed through qPCR analysis (Supplemental Figure 2A). Subsequently, we examined the effect of *Aebp1* silencing on osteogenic differentiation and mineralization processes in osteoblasts. In *Aebp1*-KD groups, a consistent reduction was observed in the mRNA expression levels of key osteogenic markers, including *Sp7*, *Col1a1*, and *Runx2* (Supplemental Figure 2B). Western blot analysis further corroborated these findings, revealing decreased protein levels of ACLP, Col1a1, RUNX2, and osteocalcin (OCN) in *Aebp1*-KD osteoblasts (Supplemental Figure 2C). Functional assays demonstrated a significant reduction in alkaline phosphatase (ALP) activity, coupled with a complete abrogation of mineralized nodule formation, as evidenced by Alizarin Red S (ARS) staining in the *Aebp1*-KD group (Supplemental Figure 2, D and E). Concordantly, impaired osteoblast differentiation was observed in osteoblasts isolated from *Aebp1^OsxCre^* mice (Supplemental Figure 2F), as well as in osteoblasts derived from *Aebp1^fl/fl^* mice transduced with adenovirus expressing Cre recombinase (Ad-Cre) (Supplemental Figure 2, G and H). These findings collectively underscore the critical role of *Aebp1* in bone formation, highlighting its indispensable function in regulating osteoblast differentiation and maturation.

Osteocytes, the terminally differentiated form of osteoblasts, are embedded in bone matrix and mediate mechanosensing and intercellular communication via dendritic processes. Abnormal dendritic architecture can compromise mechanotransduction and bone integrity. To assess effects of *Aebp1* deletion on osteocyte morphology, we stained cortical sections with phalloidin in *Aebp1^OsxCre^* mice and littermate controls. The results reveal a significant reduction in both the number and length of dendrites in osteocytes from *Aebp1^OsxCre^* mice. These data are consistent with disrupted osteocyte connectivity in *Aebp1*-deficient Osx-lineage bone, which may impair mechanosensing and structural integrity.

**Figure 5:**
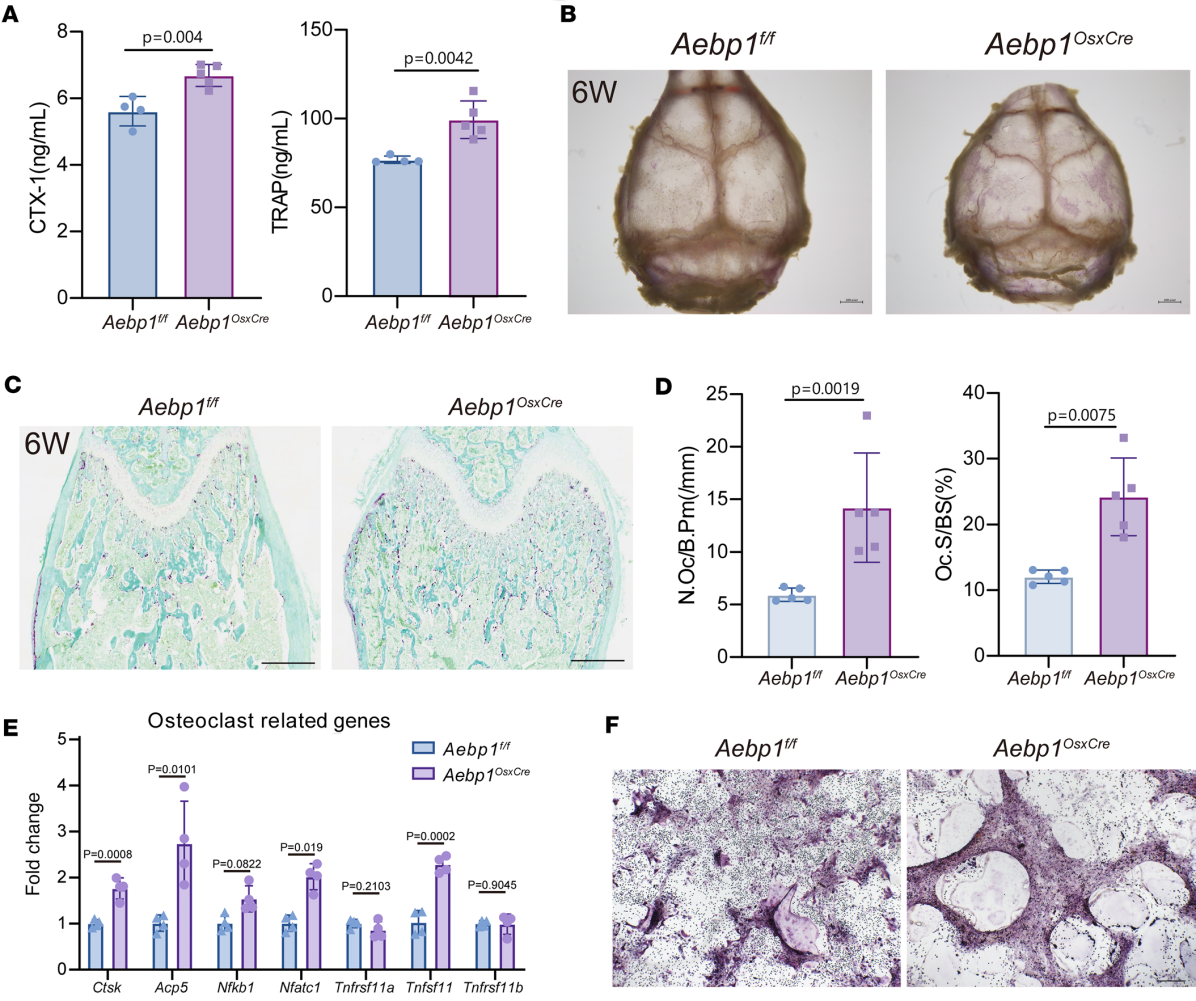
Loss of *Aebp1* in mouse osteoblasts led to increased osteoclasts activity. (**A**) Quantitative analysis of CTX-1 and TRAP in serum littermate mice of the indicated genotypes at 6 weeks old (*n* ≥ 4 for each genotype, Student’s *t* test, data shown as mean ± SD). (**B**) Representative photograph of whole-mount TRAP staining of skull from 6-week-old mice. Scale bar: 2 mm (**C**) Representative TRAP staining of femurs from 6-week-old mice. Scale bar: 200 μm. (**D**) Quantitative analysis of TRAP staining of **C** (*n* = 5 for each genotype, Student’s *t* test, data shown as mean ± SD). (**E**) Related expression of osteoclast related gene *Ctsk, Acp5, Nfkb1, Nfatc1, Tnfrsf11a, Tnfsf11*, and *Tnfrsf11b* in 6-week-old mice femur were assessed by qPCR (*n* = 4, Student’s *t* test, data shown as mean ± SD). (**F**) Representative image of TRAP staining of osteoclast in the osteoblast-osteoclast coculture system. Scale bar: 100 μm.

Given the coupling of osteoblast and osteoclast lineages, we asked whether *Aebp1* loss in osteoprogenitor cells indirectly influences osteoclastogenesis. To test this, we first assessed bone resorption activity by ELISA in *Aebp1^OsxCre^* mice. Serum ELISA showed significantly higher levels of Type I Collagen Cross-Linked C-Telopeptide (CTX-1) and tartrate-resistant acid phosphatase (TRAP) in the *Aebp1^OsxCre^* mice than in littermate controls (Figure 5A), indicating increased bone resorption activity. We then quantified osteoclasts by TRAP staining (Figure 5, B–D). Whole-mount TRAP staining of the skull revealed a larger TRAP^+^ area in the *Aebp1^OsxCre^* skull compared with the controls (Figure 5B). Histological analysis of the long bone confirmed a significant increase in both the number of osteoclasts per bone perimeter (N.Oc/B.Pm) and the osteoclast surface area normalized to BS (Oc.S/BS) in *Aebp1^OsxCre^* mice (Figure 5D). These findings strongly suggest enhanced osteoclast activity in the *Aebp1^OsxCre^* bone. To further investigate the mechanisms underlying this phenomenon, we analyzed the expression of osteoclast-related genes. The results show significant upregulation of *Ctsk*, *Acp5*, *Nfatc1*, and *Tnfsf11* in *Aebp1^OsxCre^* mice, while the expression levels of *Tnfrsf11a* and *Tnfrsf11b* remained unchanged (Figure 5E). Additionally, in osteoblast-osteoclast coculture system, osteoblasts derived from *Aebp1^OsxCre^* mice exhibited an enhanced ability to support osteoclastogenesis, as evidenced by the increased formation of giant multinucleated TRAP^+^ osteoclasts (Figure 5F). Taken together, these findings demonstrate that *Aebp1* deletion in osteoblasts not only impairs osteoblast differentiation and maturation and may indirectly enhances osteoclast differentiation. This dual effect contributes to severe defects in bone remodeling, with the combined effect of diminished osteoblast function and increased osteoclast activity contributing to the skeletal abnormalities observed in *Aebp1^OsxCre^* mice.

### Aebp1 supports canonical Wnt/β-catenin signaling in the developing bone.

**Figure 6:**
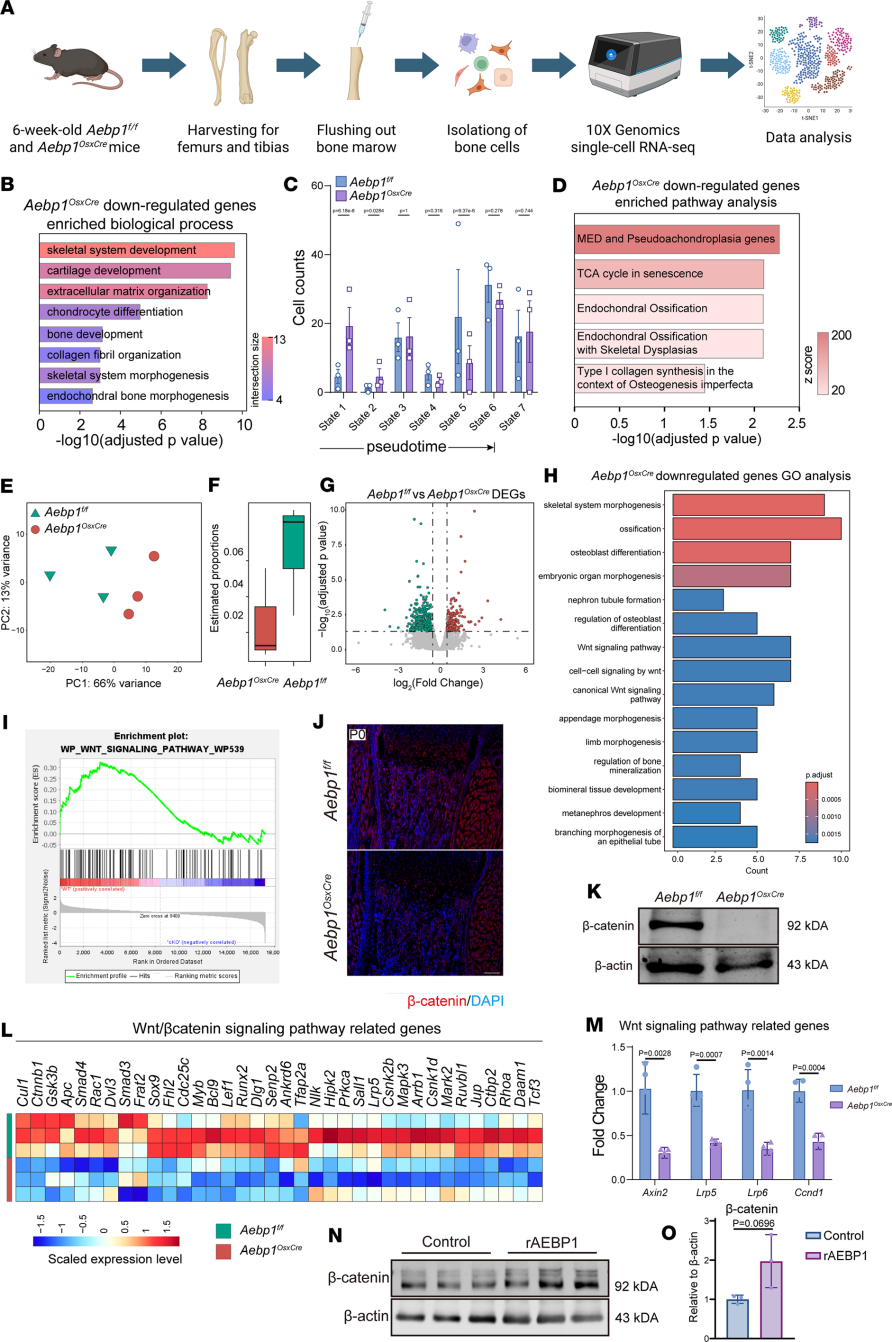
*Aebp1* deletion leads to reduction in Wnt/β-catenin signaling activity in the developing long bones. (**A**) Schematics of isolating 6-week-old hindlimb bones for the scRNA-Seq analysis. (**B**) Results of enriched biological processes in top 100 downregulated DEGs of *Aebp1^OsxCre^* osteoblasts. (**C**) Cell counts of *Aebp1^fl/fl^* and *Aebp1^OsxCre^* osteoblasts among different pseudotime states. (**D**) Results of enriched pathways in top 100 downregulated DEGs of *Aebp1^OsxCre^* osteoblasts. (**E**) Principal component analysis (PCA) of the bulk RNA-Seq data of *Aebp1^fl/fl^* and *Aebp1^OsxCre^* femoral samples (*n* = 3 for each genotype). (**F**) Estimated proportions of cluster S1 in bulk RNA-Seq data calculated by deconvolution algorithm. (**G**) Volcano plot exhibits the DEGs of the *Aebp1^OsxCre^* group compared with the *Aebp1^fl/fl^* group (*n* = 3 for each genotype). Green dots show genes more highly expressed in the *Aebp1^fl/fl^* group. Red dots show genes more highly expressed in the *Aebp1^OsxCre^* group. (**H**) GO enrichment analysis of DEGs downregulated in the *Aebp1^OsxCre^* group. (**I**) GSEA shows the enrichment score of Wnt signaling pathway by comparing with the *Aebp1^OsxCre^* group to the *Aebp1^fl/fl^* group. (**J**) Representative images of β-catenin IF staining of the humerus sections from P0 pups with the indicated genotypes. Scale bar: 100 μm. (**K**) Western blotting analyses of the femur bone tissue lysates of the P0 pups with indicated genotypes. (**L**) Scaled expression levels of selected Wnt pathway–related genes among *Aebp1^fl/fl^* and *Aebp1^OsxCre^* groups was shown by heatmap. (**M**) Related expression of Wnt signaling pathway related gene Axin2, Lrp5, Lrp6, and Ccnd1 in P0 mice femur were accessed by qPCR (*n* = 4, Student’s *t* test, data shown as mean ± SD). (**N**) Western blotting analyses of the β-catenin protein level of MC3T3-E1 cells. (**O**) Quantification of the Western blotting results in **N** (*n* = 3, Student’s *t* test, data shown as mean ± SD).

To investigate how *Aebp1* loss (*Aebp1^OsxCre^*) impairs osteoblast differentiation, we performed single-cell transcriptomic RNA-Seq (scRNA-Seq) on humeral and femoral single-cell samples from littermate pair mice (*n* = 4 per group) (Figure 6A). Quality control was performed separately for each scRNA-Seq dataset (Supplemental Figure 4A). Canonical osteoblastic differentiation markers, including *Alpl*, *Col1a1*, *Sp7*, *Bglap*, and *Ibsp*, were used to identify osteoblastic lineage subpopulations among unsupervised analyzed clusters (Supplemental Figure 4B). These subpopulations were subsequently subset and integrated after batch-effects correction for downstream analyses (Supplemental Figure 4, C and D). Osteoblast cell populations were comprehensively identified and validated by sequential marker gene analysis, Gene Ontology (GO) enrichment analysis, and scoring (Supplemental Figure 4, C–F). The downregulated genes in *Aebp1^OsxCre^* osteoblasts were enriched in the biological process including cartilage development, chondrocyte differentiation, bone development, endochondral ossification, and type I collagen formation (Figure 6, B and D). These findings highlight the disrupted osteoblastic transcriptomic features and impaired differentiation caused by *Aebp1* deletion.

We also examined osteocyte and chondrocyte related signals. Osteocyte markers (*Sost, Dmp1, Phex, Fam20c, Pdpn, Gja1, Dkk1*) showed cluster-restricted patterns, and WT-enriched GO terms highlighted osteocyte functions (Supplemental Figure 4, G and H). Chondrocyte marker–positive cells were scarce. Nevertheless, their distribution and WT-enriched cartilage GO terms are consistent with low-abundance cartilage programs (Supplemental Figure 4, I and J).

To further investigate the differentiation trajectory of osteoblasts, we next performed pseudotime analysis. Cells were ordered into 7 states (Supplemental Figure 5, A and B). Notably, *Aebp1^OsxCre^* cells were enriched in states 1–3, whereas WT cells predominated in states 4–6 (Figure 6C and Supplemental Figure 5C). Expression analysis of state-specific osteoblast markers supported the differentiation trajectory of osteoblast lineage subpopulations progress from state 1 to state 6 (Supplemental Figure 5D). These findings suggest that *Aebp1* KO may lead to differentiation arrest in osteoblasts, preventing their progression along the normal trajectory.

We then queried *Csf1r* and *Tnfrsf11a* to identify osteoclast progenitors. Cluster 21 showed enriched expression of *Csf1r* and *Tnfrsf11a* (Supplemental Figure 6A). GO analysis revealed that this cluster is primarily associated with functions related to the assembly of the major histocompatibility complex (MHC) (Supplemental Figure 6B). Based on these findings, we annotated this population as *Csf1r^+^* and *Tnfrsf11a*^+^ monocytes. Significantly upregulated genes in *Aebp1^OsxCre^* *Csf1r*^+^*Tnfrsf11a*^+^ monocytes were enriched in Type I IFN–related (IFN-I–related) pathways (Supplemental Figure 6C). Notably, mRNA expression of IFN-I signaling pathway–related genes, including *Bst2, Irf7, Irf9, Ifit1, Ifit2, Isg15*, and *Mx2*, were significantly increased in *Aebp1^OsxCre^* mice (Supplemental Figure 6D). These results suggest that activation of the IFN-I signaling pathway may contribute to the increased osteoclast population observed in the long bones of *Aebp1^OsxCre^* mice.

To complement the lower per-cell coverage of scRNA-Seq, we performed bulk transcriptome RNA-Seq (bulk RNA-Seq) on humeral and femoral samples from littermate pair mice (*n* = 3 per group) using identical tissue preparation protocols. Principal component analysis (PCA) revealed distinct gene expression profiles between the control (*Aebp1^fl/fl^*) and *Aebp1^OsxCre^* mice (Figure 6E). Deconvolution analysis indicated a higher proportion of osteoblasts in the humeral and femoral samples of *Aebp1^fl/fl^* mice compared with *Aebp1^OsxCre^* mice (Figure 6F). Differential expression analysis, visualized via a volcano plot, identified differentially expressed genes (DEGs) between the 2 groups (Figure 6G). GO analysis revealed a downregulation of genes involved in skeletal system formation, ossification, osteoblast differentiation, and Wnt signaling pathways in the *Aebp1^OsxCre^* mice (Figure 6H). Gene Set Enrichment Analysis (GSEA) showed Wnt-related gene sets enriched in *Aebp1^fl/fl^* mice compared with *Aebp1^OsxCre^* mice (Figure 6I). A heatmap of gene expression levels highlighted a reduced expression of canonical Wnt signaling-related genes in *Aebp1^OsxCre^* mice compared with *Aebp1^fl/fl^* mice (Figure 6L). In addition to the humeral and femoral samples, cranial tissue from the same cohort of mice was subjected to bulk RNA-Seq (Supplemental Figure 6A). Clustering analysis and PCA confirmed distinct transcriptomic profiles consistent with group classifications (Supplemental Figure 6, B and C). The expression of the *Aebp1* gene in *Aebp1^OsxCre^* mice was significantly lower compared with that in *Aebp1^fl/fl^* mice (Supplemental Figure 6D). GO analysis revealed a reduction in the expression of genes associated with ossification, bone development, and osteoblast differentiation in *Aebp1^OsxCre^* mice (Supplemental Figure 6, E and F). Canonical osteoblast differentiation marker genes, including *Alpl*, *Sp7*, *Runx2*, *Col1a1*, and *Ibsp*, were significantly downregulated in *Aebp1^OsxCre^* mice (Supplemental Figure 6G). Similarly, genes related to Wnt signaling, such as *Ctnnb1*, *Tcf7*, *Lrp5*, and *Fzd5*, exhibited markedly diminished expression in *Aebp1^OsxCre^* mice (Supplemental Figure 6H).

Given the critical roles of the Wnt/β-catenin signaling pathway in bone development, our study focused on this pathway. β-Catenin, a pivotal signal transducer in the Wnt/β-catenin signaling pathway, was markedly reduced in P0 long bones of *Aebp1^OsxCre^* mice (Figure 6, J and K). Additionally, mRNA expression levels of *Axin2* and *Ccnd1*, the transcriptional readouts of canonical Wnt pathway, were reduced, and Wnt pathway components *Lrp5* and *Lrp6* were also lower in *Aebp1^OsxCre^* mice (Figure 6M). To further investigate the role of ACLP in the Wnt/β-catenin signaling pathway, recombinant ACLP was introduced into the MC3T3-E1 cell line. Western blot analysis demonstrated an increase in β-catenin protein levels in cells treated with recombinant ACLP compared with the control group (Figure 6, N and O). Together, these findings suggest that ACLP is required to sustain baseline Wnt/β-catenin signaling in developing bone.

### Enhanced Wnt/β-catenin signaling restores bone formation in Aebp1 mutants.

**Figure 7:**
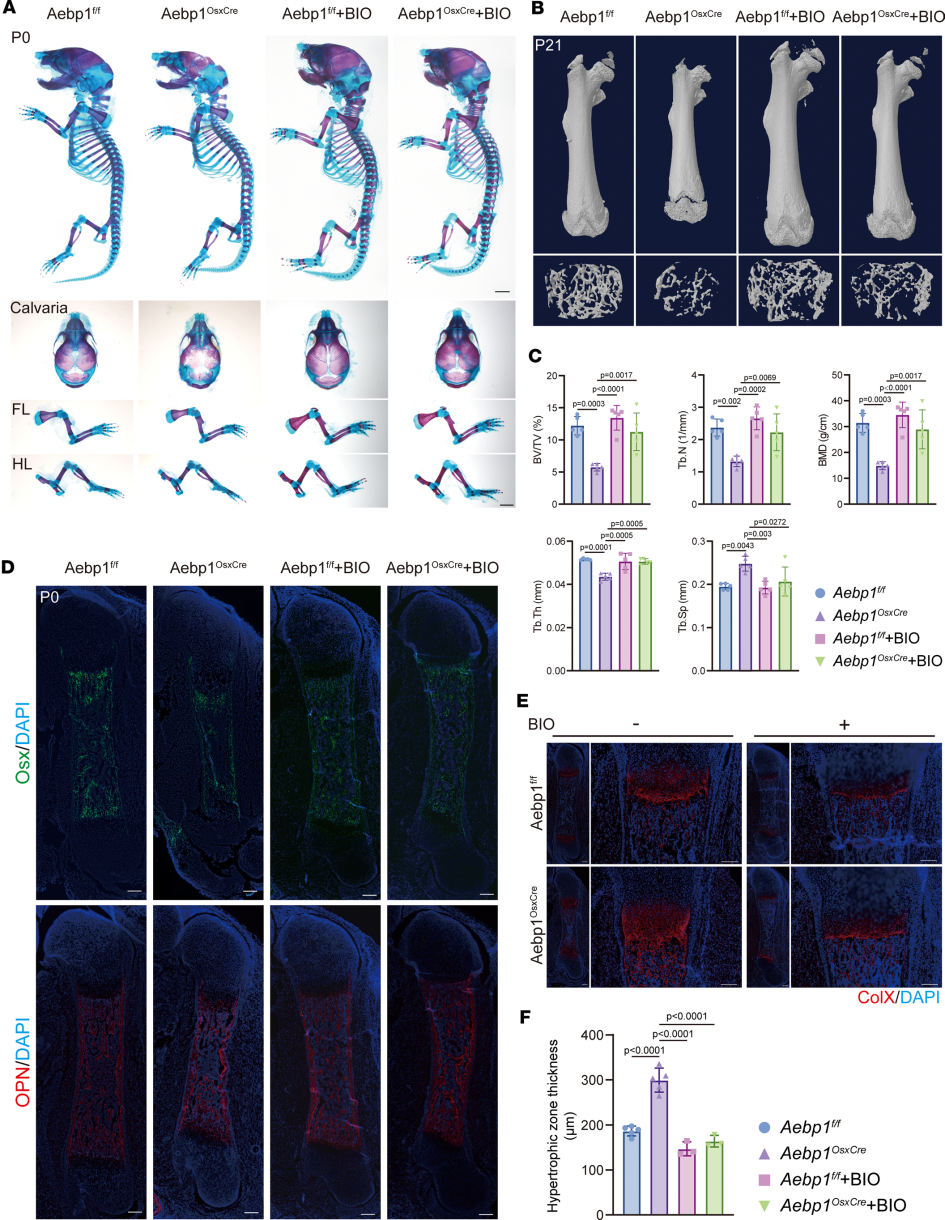
Skeleton defects in *Aebp1^OsxCre^* mice were rescued by BIO treatment. (**A**) Whole-mount Alizarin red and Alcian blue staining of littermate mice of the indicated genotypes at P0. The calvaria, forelimb and hindlimb were shown at the bottom. Scale bar: 1 mm. (**B**) Representative μCT images of femurs from 3-week-old littermate mice with the indicated genotypes. (**C**) Quantification of indicated parameters of μCT scanning (*n* = 5 for each genotype, 1-way ANOVA, data shown as mean ± SD). (**D**) Representative images of Osx (top) and OPN (bottom) IF staining of the femur sections from P0 pups with the indicated genotypes. Scale bar: 200 μm. (**E**) Representative images of type X collagen IF staining of the femur sections from P0 pups with the indicated genotypes. Scale bar: 200 μm. (**F**) Quantification of hypertrophic zone thickness of the femur sections from P0 pups with the indicated genotypes (*n* ≥ 3, Student’s *t* test, data shown as mean ± SD).

To test whether boosting Wnt/β-catenin signaling rescues the phenotype, we pharmacologically potentiated this pathway. Pregnant females were treated with BIO, a natural inhibitor of GSK3β that stabilizes β-catenin (24–26), thereby enhancing Wnt/β-catenin signaling in the developing embryos. BIO treatment substantially ameliorated skeletal abnormalities and augmented bone formation in *Aebp1^OsxCre^* mice at P0 and P21 (Figure 7, A and B). Additionally, μCT analyses corroborated the restoration of trabecular bone mass in adult *Aebp1^OsxCre^* mice (Figure 7, B and C).

IF staining revealed substantial reinstatement of Osx and OPN expression levels at P0 (Figure 7D). Furthermore, BIO treatment increased osteoblast numbers (Supplemental Figure 8, A and B) and cortical bone thickness (Supplemental Figure 8, C and D), and it concurrently mitigated osteoclast hyperactivity in *Aebp1^OsxCre^* mice at P21 (Supplemental Figure 8, E–G). Notably, in WT mice, the same BIO regimen elicited no discernible alterations on basal bone formation. ARS staining demonstrated that BIO treatment restored mineralization defects in *Aebp1^OsxCre^* mice (Supplemental Figure 9A). During osteogenic differentiation, BIO-treated *Aebp1^OsxCre^* osteoblasts exhibited elevated transcriptional levels of key osteoblast differentiation markers, including *Bglap, Col1a1, Runx2*, and *Sp7* (Supplemental Figure 9B). Collectively, these findings underscore the efficacy of BIO in ameliorating osteoblast differentiation deficits induced by Aebp1 ablation.

Moreover, BIO treatment significantly reduced the Col10a1^+^ hypertrophic chondrocyte region in *Aebp1^OsxCre^* long bones toward WT levels (Figure 7, E and F). Taken together, these results indicate that the *Aebp1* loss in osteoprogenitor cells compromises bone formation by attenuating Wnt/β-catenin signaling. Importantly, pharmacologic reinforcement of Wnt/β-catenin signaling may represent a potential strategy to mitigate clEDS2-related skeletal defects, although clinical translation remains to be determined.

## Discussion

Mutations in the human *AEBP1* gene have been associated with the genetic etiology of clEDS2 (7–10, 13). Patients with clEDS2 exhibit abnormalities in the skeletal system (27–30). ACLP (encoded by *AEBP1*) is expressed in collagen-rich tissues, including bone, suggesting a role in skeletal physiology (14, 31). Consistent with this, our study demonstrated that the ablation of *Aebp1* function in mice osteoprogenitor cells produced clEDS2-like skeletal abnormalities, including reduced bone mass, shorter body length, and structural defects. Mechanistically, these defects were attributed to impaired osteoblast differentiation caused by the downregulation of Wnt/β-catenin signaling. Remarkably, treatment with the GSK3 inhibitor BIO restored Wnt signaling and ameliorated the skeletal defects in *Aebp1*-deficient mice, highlighting the critical role of ACLP in maintaining Wnt/β-catenin signaling for normal skeletal development.

Our findings align with previous reports identifying AEBP1 as a molecular marker of the osteoblast lineage, with its expression upregulated during osteogenic differentiation (32, 33). ACLP was observed to colocalize with Osx^+^ cells and OPN (Figure 1, A and B), consistent with production in osteoblasts. While global KO studies have revealed embryonic lethality due to abdominal fissures and curled tails, the precise skeletal phenotype of ACLP-deficient mice has remained unclear due to the widespread expression of ACLP in tissues such as muscles and ligaments (31, 33). By employing conditional KO models, we specifically ablated *Aebp1* in targeted cell populations, enabling us to uncover its role in skeletal development. These mice exhibited reduced body length, diminished bone mass, and structural abnormalities (Figure 2), paralleling the skeletal defects observed in patients with clEDS2.

Endochondral ossification necessitates the initial differentiation of mesenchymal stem cells into chondrocytes, followed by their proliferation and hypertrophy. This is succeeded by blood vessel invasion, during which mesenchymal cells differentiate into osteoblasts, ultimately forming bone tissue (19, 34). In murine models, the primary ossification center of long bones begins to form at E15.5, whereas the secondary ossification center appears 5–7 days postnatally and is fully developed by P21 (35). Our study demonstrates that the absence of *Aebp1* led to delayed endochondral ossification. Specifically, *Aebp1^OsxCre^* mice showed an expanded Col10a1^+^ hypertrophic zone at P0 and persistent Safranin O^+^ cartilage within marrow and secondary ossification centers at P3 and P21, indicating delayed endochondral ossification (Figure 3). In addition to osteoblasts, Osx expression has been reported in subsets of growth-plate chondrocytes (chondroblasts and hypertrophic chondrocytes), and *OsxCre* can recombine a fraction of chondrocytes (36, 37). This plausibly contributes to the growth plate–related effects observed in our *Aebp1^OsxCre^* mice and informs interpretation of the endochondral phenotypes. Single-cell pseudotime analysis revealed enrichment of *Aebp1*-deficient osteoblastic lineage cells in earlier differentiation states (state 1–3) and depletion of later differentiation states (state 4–6), consistent with a differentiation block. Moreover, deletion of *Aebp1* in osteoprogenitor cells resulted in reduced expression of key osteogenic markers such as *Sp7, Runx2, Col1a1*, and *Bglap*. This was accompanied by diminished mineralized nodule formation and ALP activity, indicating impaired osteogenic differentiation. Histomorphometric analysis further supported these findings, showing reduced osteoblast numbers, BFR, and MAR in *Aebp1*-KO mice. The impairment in osteoblast differentiation due to *Aebp1* deletion, evidenced by the diminished expression of markers such as Osterix and OPN, highlights the critical association between Wnt signaling and osteoblast activity (19, 21). These findings align with the essential role of Wnt signaling in regulating osteoblast function and bone formation (38–41).

Interestingly, *Aebp1* deletion significantly affected osteocyte morphology, characterized by a reduction in dendrite number and length. This impairment compromises osteocytes’ mechanosensing ability and intercellular communication, which are critical for maintaining bone homeostasis. As the primary mechanosensory cells in bone, osteocytes detect mechanical strain and coordinate bone remodeling by interacting with osteoblasts and osteoclasts through their dendritic processes. These processes form a lacunocanalicular network essential for signaling molecule transport and nutrient exchange (42–44). The observed reduction in dendrite number and length in *Aebp1^OsxCre^* mice suggests a diminished capacity to sense mechanical forces, thereby disrupting bone remodeling and skeletal integrity. scRNA-Seq revealed that the S3 cluster within the osteoblastic lineage cells exhibited high expression of osteocyte-related genes. GO analysis further indicated a downregulation of “regulation of synapse organization” and “regulation of synapse structure and activity” in *Aebp1^OsxCre^* mice, suggesting impaired synaptic morphology and function in osteocytes. Additionally, osteocyte dendrites are essential for the production and regulation of sclerostin (SOST), a key inhibitor of Wnt/β-catenin signaling. SOST secretion modulates osteoblast activity and bone formation, particularly under reduced mechanical loading conditions (45). The disruption of dendritic morphology in *Aebp1^OsxCre^* mice likely leads to dysregulated SOST production, further impairing Wnt/β-catenin signaling and osteoblast differentiation. However, this hypothesis requires further experimental validation. Consistent with this interpretation, scRNA-Seq showed cluster-restricted osteocyte markers with WT-enriched osteocyte GO terms, whereas chondrocyte-like cells were scarce and exhibited limited signal strength (Supplemental Figure 4, G–J).

Bone modeling throughout development is a dynamic process characterized by continuous metabolic activity, referred to as bone remodeling. This process encompasses both the formation of bone by osteoblasts and its resorption by osteoclasts. The equilibrium of bone remodeling is essential for sustaining bone homeostasis, and understanding the regulation of this balance is potentially pivotal for the treatment of various bone diseases (46). Disruptions in the equilibrium between osteoblastic and osteoclastic activities can result in bone disorders (47–49). In this study, we present evidence demonstrating the critical role of ACLP in osteoblasts in the regulation of bone metabolism. Serum analysis of mice deficient in *Aebp1* expression in osteoblasts revealed increased markers indicative of bone resorption activity (Figure 5A). Concurrently, histological staining showed enhanced osteoclast activity in the bone tissue of these mice (Figure 5, B–D). Furthermore, the expression of *Ctsk,* *Acp5, Nfatc1*, and *Tnfsf11* was elevated in bone from *Aebp1^OsxCre^* mice (Figure 5E). Coculture experiments demonstrated that osteoclast differentiation was significantly enhanced when osteoclasts were cocultured with *Aebp1^OsxCre^* osteoblasts (Figure 5F). These findings suggest that the loss of *Aebp1* in osteoblasts leads to increased osteoclast activity. Notably, the expression levels of OPG and RANK remained unchanged (Figure 5E), indicating that other mechanisms may underlie these observations. Further analysis of relevant references may help identify potential reasons for this phenomenon. To explore potential regulatory mechanisms, we analyzed scRNA-Seq data. Because mature osteoclasts are large and multinucleated, they are typically undercaptured by droplet-based scRNA-Seq platforms. Instead, we identified a population of mononuclear cells marked by *Csf1r* and *Tnfrsf11a*, a phenotype consistent with established markers of osteoclast-lineage precursors (50, 51). Comparative analysis revealed that *Csf1r*^+^*Tnfrsf11a*^+^ mononuclear cells in *Aebp1^OsxCre^* mice showed significant upregulation of genes enriched in IFN-I pathways. IFN-I pathways modulate osteoclastogenesis in a context-dependent manner: IFN-β provides negative feedback that restrains RANKL-driven differentiation (52, 53), and tonic IFN-I signaling can restrict osteoclast fusion and inflammatory bone loss (54, 55). Nonetheless, IFN-I–associated transcriptional programs are frequently observed in inflammatory bone remodeling. Thus, our data raise the hypothesis that an IFN-I–primed precursor state may contribute to the increased osteoclast indices in *Aebp1^OsxCre^* bone, and this will require functional testing.

Bone development is regulated by distinct transcriptional networks and signaling pathways, including TGF-β, STAT3, and β-catenin. Among these, the Wnt/β-catenin signaling pathway plays a pivotal role in bone development. Functional impairment of β-catenin leads to severe defects in long bone and craniofacial structures, with symptoms such as craniofacial malformations, limb development failure, cleft palate, and pelvic hypoplasia (56–61). In *Aebp1^OsxCre^* mice, reduced bone mass in long bones and cranial defects closely resemble the phenotypes of β-catenin-KO mice. RNA-Seq analysis further reveals diminished activity of the Wnt/β-catenin signaling pathway in the long bones and skulls of these mice, characterized by significant reductions in β-catenin expression and Wnt pathway–related gene expression (Figure 6, J–M). In vitro experiments demonstrate that recombinant ACLP increased β-catenin levels (Figure 6, M and N), supporting a link between ACLP and canonical Wnt activity in osteoblasts. ACLP has also been identified as a liver-expressed ligand that activates the canonical Wnt pathway and exacerbates nonalcoholic steatohepatitis (NASH) pathology (12), further highlighting the role of *Aebp1* in amplifying Wnt/β-catenin signaling across different biological contexts. Therapeutically, GSK3 inhibition with BIO improved bone mass, normalized osteoblast numbers, and reduced osteoclast indices in *Aebp1^OsxCre^* mice. BIO is an ATP-competitive GSK3α/β inhibitor with high selectivity versus most kinases in profiling assays, consistent with its use as a tool compound to stabilize β-catenin in vivo (24, 25). Notably, BIO treatment in WT mice does not significantly alter bone mass, suggesting that Wnt signaling modulation may be most effective under pathological conditions or signaling deficits (62–64). These findings emphasize the therapeutic potential of GSK3 inhibitors in conditions such as clEDS2, where targeted stabilization and activation of β-catenin can address skeletal abnormalities. Moreover, GSK3 inhibitors have been extensively studied in other diseases, including neurodegenerative disorders, demonstrating their broad therapeutic potential (65). Recent advancements in elucidating the molecular mechanisms of Wnt signaling have underscored the potential for targeting specific components of this pathway to achieve more precise therapeutic interventions. Such strategies may be particularly advantageous in the management of conditions like clEDS2 (62). Additionally, exploring alternative pathways that interact with Wnt/β-catenin could provide new therapeutic targets and enhance the efficacy of existing treatments.

Finally, these findings should be interpreted in light of several limitations. Our study was designed to interrogate bone-intrinsic mechanisms by deleting *Aebp1* in the Osx-lineage. We therefore did not systematically assess extraskeletal EDS manifestations such as joint laxity and cardiovascular involvement. These phenotypes will likely require models targeting tendon/ligament or vascular smooth muscle (*ScxCre* and *Myh11Cre*). In addition, for P21 and older cohorts, we primarily analyzed male mice to minimize sex-hormone variability, which may limit generalizability. This need adequately powered and sex-balanced studies. We also did not detect overt scoliosis in our colony, but this absence should be interpreted cautiously because the current bone-focused model does not directly probe soft-tissue contributions to axial deformity. Finally, direct comparison with global *Aebp1*-KO animals is constrained by embryonic and systemic consequences in whole-body mutants. Thus, phenotype differences arising from non–cell-autonomous effects fall outside the scope of the present Osx-restricted approach.

In summary, our study elucidates *Aebp1* as a critical regulator of skeletal development through its modulation of Wnt/β-catenin signaling. The loss of *Aebp1* in osteoprogenitor cells disrupts osteoblast differentiation, delays ossification, and enhances osteoclast activity, culminating in severe bone remodeling defects. Pharmacological activation of Wnt/β-catenin signaling using BIO effectively restores bone formation in *Aebp1*-KO mice, offering a promising therapeutic approach for clEDS2-related skeletal disorders. These findings provide insights into the molecular mechanisms underlying *Aebp1*-mediated bone development and highlight potential avenues for targeted treatments of clEDS2.

## Methods

### Sex as a biological variable.

In postnatal studies, newborn cohorts included both sexes, and for P21 and older cohorts, we analyzed males only to minimize sex hormone–related variability.

### Mice.

*Aebp1^fl/fl^* mice bearing loxP sites flanking exons 10–20 of the *Aebp1* gene were purchased from the Shanghai Model Organisms Center Inc. *Osx1-GFP:Cre* (*OsxCre*, stock No. 006361) strain was a gift from Xiaochun Bai (Department of Cell Biology, School of Basic Medical Science, Southern Medical University, Guangzhou, China). All animals were maintained in the animal facility of Southern Medical University and housed under standard conditions of constant temperature and humidity on a 12/12-hour light/dark cycle. To generate littermate cohorts, we used *OsxCre*; *Aebp1^fl/+^* (male) crossed with *Aebp1^fl/fl^* (female). Representative data from analyses of a minimum of 3 control and mutant littermates in each experiment are shown.

### Histology and IF staining.

Embryos and early postnatal tissues were fixed at 4°C overnight in 4% paraformaldehyde (PFA) in phosphate-buffered saline (PBS) and processed for either cryostat or paraffin sections. Tissue sections were used for H&E staining, Safranin O staining, Von Kossa staining, Masson’s trichrome staining, and TRAP staining according to the standard protocol. For IF staining, sections were rehydrated, permeabilized with PBST (PBS + 0.05% Triton), and blocked in 10% donkey/goat serum in PBST. IF staining was performed using standard methods, and details of primary and secondary antibodies are provided in Table 1. Sections were mounted in mounting medium containing nuclear stain DAPI from Vector laboratories (catalog. H-1200). Images were acquired with Zeiss Axio Imager D2 (Zeiss, Germany).

### Cell culture.

Mice calvarial osteoblasts were isolated from 3-day-old neonatal mice by collagenase and dispase II digestion (66) and cultured in α-minimum essential medium (α-MEM) containing 10% FBS and 1% penicillin and streptomycin (P/S, all from Gibco) in a 37°C incubator with a 5% CO_2_ atmosphere. Once adherent cells reached 80% confluence, they were digested and cultured further. Cells were passaged to the third generation before proceeding with subsequent experiments.

The MC3T3-E1 Subclone 14 cell line (no. CL-0325) was purchased from Procell Life Science & Technology Co. Ltd. The MC3T3-E1 cells were cultured in α-MEM containing 10% FBS, and 1% P/S (Gibco, Grand Island, NY, USA).

For osteogenic induction, mice calvarial osteoblasts and MC3T3-E1 cells were cultured in osteogenic medium containing 50 μg/mL ascorbic acid and 10 mM β-glycerophosphate, and then subjected to ALP staining on day 7 and ARS staining on day 14.

### siRNA-mediated KD and cell transfection.

*Aebp1*-specific siRNAs (sequences in Table 2) and negative control siRNA (NC) (RiboBio, Guangzhou, China) were used for transfection. Transfection of siRNA oligonucleotides was performed using Lipofectamine RNAimax (Invitrogen, Carlsbad, CA, USA) according to the manufacturer’s instructions. *Aebp1* expression was determined by quantitative reverse transcription PCR (RT-PCR). Transfected cells were passaged and used for downstream analyses.

### Adenovirus construction and cell transfection.

The Cre recombinase or eGFP adenovirus (~10^12^ pfu/mL) (HanBio Technology, Shanghai, China) was diluted 1:2,000 to infect cells in vitro. After 4 hours, fresh medium was added. After 24 hours, the medium was changed.

### ALP and ARS staining.

For ALP staining, differentiated osteoblasts and MC3T3-E1 cells were fixed with 4% paraformaldehyde (Solarbio, China) for 15 minutes. The cells were washed 3 times with PBS and stained with 1-Step nitro blue tetrazolium (NBT)/5-bromo-4-chloro-3-indolyl phosphate (BCIP) (Thermo Fisher, MA, USA) for 30 minutes and washed by PBS. ALP^+^ cells were visualized by light microscopy or scanning.

For ARS staining, differentiated osteoblasts and MC3T3-E1 cells were fixed with 4% paraformaldehyde for 15 minutes. The cells were washed 3 times with distilled water and stained with ARS staining solution for 15 minutes. The staining solution was removed, and the cells were washed 3 times in distilled water. The mineralized part was visualized by scanning.

### Skeletal preparation.

The protocol for Alcian blue staining for cartilage and ARS staining for mineralized tissues was described before (67). In brief, mice were eviscerated and the skin was removed, and the resulting samples were transferred into acetone for 48 hours after overnight fixation in 100% ethanol. Skeletons were then stained in Alcian blue and ARS solution and sequentially cleared in 1% KOH/20% glycerol. The staining was photographed with N23977 Stereomicroscopy (Zeiss, Germany).

### μCT scanning analyses.

μCT scanning analyses were performed on a SkyScan 1276 system. Mouse hind limbs and skulls were harvested, soft tissues were removed, and the remaining tissues were stored in 70% ethanol prior to scanning. For femoral trabecular bone analysis, the volume of interest (VOI) was defined as a 1.0 mm-high region beginning 0.5 mm proximal to the distal growth plate. Scanning was conducted at 70 kVp and 200 μA using a 0.25-mm aluminum filter, with an isotropic voxel size of 7 μm, an exposure time of 350 ms, and a rotation step of 0.25°. Images were reconstructed using Gaussian smoothing set to 5 and ring artifact correction set to 8. A global threshold of 70-255 was applied for bone segmentation (68). The region of interest (ROI) was segmented and reconstructed into 3-dimensional images. The related properties of trabecular bone, such as Tb.Th, Tb.N, Tb.Sp, BV/TV, BMD, were then calculated according to distance transformation of the binarized images.

### Whole-mount TRAP histochemistry.

Calvaria from 6-week-old mice were dissected, soft tissue was removed, and the calvaria were fixed in 100% methanol for 5 minutes. Bones were thoroughly washed in PBS, and TRAP staining was performed per the instructions of the manufacturer (Sigma) (69). The staining was photographed with N23977 Stereomicroscopy (Zeiss, Germany).

### Bone histomorphometric analysis.

Three-week-old *Aebp1^fl/fl^* and *Aebp1^OsxCre^* mice were s.c. injected with 20 mg/kg Calcein (Sigma) on days 7 and 2 before euthanization, respectively. Femurs were fixed in 70% ethanol, dehydrated, and embedded in methyl methacrylate, and transverse sections were cut at 15 μm at the 50% femoral length (±5%) reference level. MAR (μm/day) was calculated as the mean interlabel distance divided by the time between labels and was scored only at double-labeled sites. BFR/BV (per 1/day) was derived as BFR/BV = MAR × (MS/BS) × (BS/BV) within the cortical ROI, where MS/BS is mineralizing surface per BS (%) (70). Measurements were performed with the OsteoMeasure Image Analyzer (OsteoMetric) under blinded conditions. Static histomorphometry (N.Ob/B.Pm, N.Oc/B.Pm, Oc.S/BS) was performed on distal femoral secondary spongiosa following ASBMR nomenclature.

### RNA isolation and quantification of mRNA expression.

TRIzol reagent (Thermo Fisher, MA, USA) was used to extract the total RNA from the cells. PrimeScript RT Reagent Kit (Takara, Otsu, Japan) was used to synthesize complementary DNA (cDNA). qPCR was performed by using an SYBR Green PCR Kit (Takara, Otsu, Japan) as directed by the manufacturer. Gene expression levels were analyzed relative to β-actin or GAPDH. The primer sequences are shown in Table 2.

### Western blotting.

Cells and bone tissues were lysed using radioimmunoprecipitation assay (RIPA) buffer (Solarbio, Beijing, China) with protease inhibitor mixture (Roche, Swiss). Total cell lysates were analyzed using Western blotting. Western blotting analyses were conducted using standard procedures. The details of the antibodies used are provided in Table 1.

### RNA-Seq and bioinformatics analysis.

Single-cell suspensions for scRNA-Seq were generated from 6-week-old mice. For each animal, both hindlimbs were collected and only the femur and tibia were used. After removal of surrounding soft tissues, both epiphyses were trimmed to excise the secondary ossification centers and growth plates, thereby excluding epiphyseal cartilage from the sample. The bone marrow cavity was flushed with sterile PBS until the effluent ran clear, minimizing hematopoietic contamination. Then the bone tissues were placed in a 6 cm² dish with 2–3 drops of DMEM to maintain moisture. The tissue is then chopped with a blade. The chopped tissue is transferred to a 50 mL centrifuge tube, where it is combined with 2 mg/mL of preheated Collagenase Type II and IV, 15 μL of CaCl_2_ solution, and 1 mg/mL DNase I, all mixed thoroughly in a 6 mL DMEM buffer. This mixture is placed on a shaker at 37°C for 20–30 minutes. Every 10 minutes, the solution is mixed with a wide-mouthed pipette, and cell clumps are observed. Once digestion is complete and no large tissue clumps remain, the solution is filtered through 70 μm and 40 μm sieves into a new 50 mL centrifuge tube, then placed on ice and centrifuged at 400g for 4 minutes. After centrifugation at 300 *g*, the supernatant is poured off, and the pellet is resuspended with 5 mL RBC Lysis buffer, mixed, lysed on ice for 7 minutes, and centrifuged again at 300*g* for 5 minutes. The supernatant is discarded, and the pellet is resuspended with 5 mL of 0.04% BSA in PBST solution, mixed, and centrifuged at 400*g* for 4 minutes. The supernatant is discarded, and the cell mass is mixed thoroughly before determining cell concentration using a Countess II FL Automated Cell Counter. The appropriate volume of cell suspension is then prepared according to 10X Genomics requirements (71).

For GEM generation and barcoding, the Transposition Mix is prepared on ice using the Chromium Next GEM Single Cell 5′ GEM Kit v2. Nuclease-free water and the single-cell suspension are added to the Master Mix, totaling 70 μL per tube, which is then dispensed into wells. Gel Beads are vortexed and centrifuged at 3000 *g*, with 50 μL aspirated and dispensed into wells, followed by 45 μL of Partitioning Oil. The assembled chip is run in a Chromium Controller X, and GEMs are aspirated and dispensed into PCR tubes on ice. These are incubated in a thermal cycler, stored at –20°C, or processed further.

Post GEM-RT cleanup using Dynabeads involves adding 125 μL Recovery Agent, removing it after 2 minutes, and preparing the Dynabeads Cleanup Mix. The mix is added and incubated for 10 minutes before placing the sample on a magnetic separator to remove the supernatant. Ethanol washes are performed, and 35.5 μL Elution Solution I is added. The sample is incubated, centrifuged at 3000 *g*, and transferred. For cDNA amplification, the cDNA Amplification Mix is prepared, added to the sample, and incubated in a thermal cycler. The cDNA is stored or processed further with SPRIselect for cleanup, involving reagent addition, ethanol washes, and elution.

V(D)J amplification from cDNA involves preparing the V(D)J Amplification 1 Reaction Mix, adding it to the sample, and cycling in a thermal cycler. Post-amplification cleanup uses SPRIselect for size selection, with ethanol washes and elution. V(D)J Amplification 2 follows a similar process, with cleanup and storage. The 5′ Gene Expression Library Construction involves adjusting sample volumes, thermal cycling, preparing Fragmentation Mix, and performing SPRIselect cleanup, followed by adaptor ligation and amplification with a thermal cycler. The final cleanup uses SPRIselect, with ethanol washes and elution.

Sequencing is performed using the NovaSeq 6000 system with a 1×50 bp configuration, yielding approximately 5 million reads per sample.

After acquiring the single-cell RNA-Seq (scRNA-Seq) matrix data, subsequent analysis is conducted in R language (version: 4.3.2) using software R Studio (version: 2024.09.0), leveraging the Seurat (version: 5.0.1) package for unsupervised analysis (72). Tasks performed with Seurat include computing cell cycle scores, analyzing mitochondrial gene expression rate, normalization, principal components analysis (PCA) based dimensionality reduction, graph-based clustering, and uniform manifold approximation and projection (UMAP) based dimensionality reduction (73). UMAP based plot is utilized for visualizing cell clusters, while bubble plots depict gene expression among cell subpopulations. Violin plots are used to display quality control parameters including number of features, number of RNA count, and percent of mitochondrial genes. Subpopulations in 8 samples with markedly expression level of canonical osteoblastic differentiation genes are screened out, subset, batch effects corrected, and integrated into one Seurat object. This integrated object undergoes another round of unsupervised analysis, with clustering results visualized via UMAP plot. The expression score of gene set of GO: 0001649 from GO database is calculated to highlight the osteoblast population in the integrated data. Marker genes are defined as genes with significantly up- or downregulated expression levels in one population compared with the others. Top 100 downregulated markers order by fold change in *Aebp1^OsxCre^* group are projected to GO analysis via platform g:Profiler (74). Similarly, different cell types were screened using distinct marker genes, including monocytes (*Tnfrsf11a*, *Csf1r*), osteocytes (*Sost*, *Dmp1*, *Phex*, *Fam20c*, *Pdpn*, *Gja1*, *Dkk1*), and chondrocytes (*Col2a1*, *Acan*, *Matn1*, *Col10a1*). DEGs between the 2 groups were selected for GO analysis.

Pseudotime trajectory analysis was performed using the Monocle2 package (75). Prior to analysis, an equal number of osteoblast cells were randomly selected from each group. All analyses were conducted using default parameters.

After acquiring the bulk RNA-Seq count data, subsequent analysis is conducted in R language using software R Studio, leveraging the DEseq2 (version: 1.42.0) package for analysis (76).

The gene count data are input, transformed, and calculated to obtained comparative data. Subsequently, GSEA software (version: 4.3.3) is employed for further GSEA, along with PCA to illustrate inter-group variability. DEGs are defined as genes with significantly up- or downregulated expression levels in one group compare to the other and showcased in volcano plots.

Top 100 downregulated DEGs order by fold change in *Aebp1^OsxCre^* group are projected to GO analysis via platform g:Profiler. Selected gene expressions are visualized using heatmaps or bar plots.

To avoid the impact of inevitable and uncontrollable cell loss in single cell preparation for scRNA-Seq, the deconvolution analysis is performed to estimate the rate of osteoblast population identified in scRNA-Seq data in bulk RNA-Seq data, conducted by the digitalDLSorteR package (version: 1.0.1) in R. The scRNA-Seq data is used as reference to generate a model for deconvolution after 70 times iteration. This model is validated through pseudo-bulk RNA-Seq and regression analyses. Next, this model is applied to bulk RNA-Seq data to calculate the estimated proportions of each population identified in scRNA-Seq data including the osteoblast population. Box plot is used to visualized the proportions of the scRNA-Seq identified osteoblast population in *Aebp1^fl/fl^* and *Aebp1^OsxCre^* group.

### Small molecule treatment.

BIO (MedChemExpress, USA) was dissolved in dimethylsulfoxide (DMSO) to 2.8 mM, before being diluted with sterile PBS to 20μM; single-use aliquots were stored at −80°C (24). The 20 μM intermediate was further diluted in sterile PBS to a 2 μM working solution for injection. Mice were injected intraperitoneally at 10 mL/kg with the 2 μM working solution. Pregnant females were dosed daily from E15.5 and postnatal mice were dosed every other day. Vehicle controls received PBS with matched residual DMSO. For cell culture, BIO was diluted directly into prewarmed culture medium to a final concentration of 3 μM. Vehicle controls received medium containing matched DMSO without BIO.

### Statistics.

Data are presented as the mean ± SD. GraphPad Prism was used to conduct the analysis. Normality was assessed by Shapiro-Wilk and homogeneity of variances by Levene’s test. Two-group comparisons used unpaired 2-tailed *t* tests. Multiple groups used 1-way ANOVA. Exact *n* and statistical tests are reported in figure legends; *P* < 0.05 was considered significant.

### Study approval.

All animal experiments were approved by the IACUC of Southern Medical University (SMUL2021136) and were following the *Guide for the Care and Use of Laboratory Animals* (National Academies Press, 2011).

### Data availability.

The single-cell RNA-Seq and bulk RNA-Seq datasets generated in this study have been deposited in the Genome Sequence Archive (GSA) under BioProject accession no. PRJCA046260 (include 2 GSA subsets CRA030157, CRA030108) and are publicly available. The analysis codes are publicly available at https://github.com/shuhaofeng/Aebp1_RNA-Seq_code; commit ID 5bb4ffe. Supporting data values for all graphs and summary data are provided in Supporting data values.

## Author contributions

SF and ZF designed research; SF, ZF, ZD, and YW performed research; SF, ZF, ZD, and YW analyzed data; LZ, ZZ, SF, ZF, ZD, YW, and SZ did investigation; SF and ZD wrote the manuscript; SF, ZD, RL, Yangchen Jin, and Yu Jin revised the manuscript; LZ and ZZ supervised the research.

## Funding support

- National Key R&D Program of China 2022YFC2502901, 2022YFC2502902 (ZZ)
- National Natural Science Foundation of China 82370886, 82572692 (LZ)
- Guangdong Basic and Applied Basic Research Foundation 2023A1515110970 (ZD)

## Supplementary Material

Supplemental data

Unedited blot and gel images

Supporting data values

## Figures and Tables

**Table 1:**
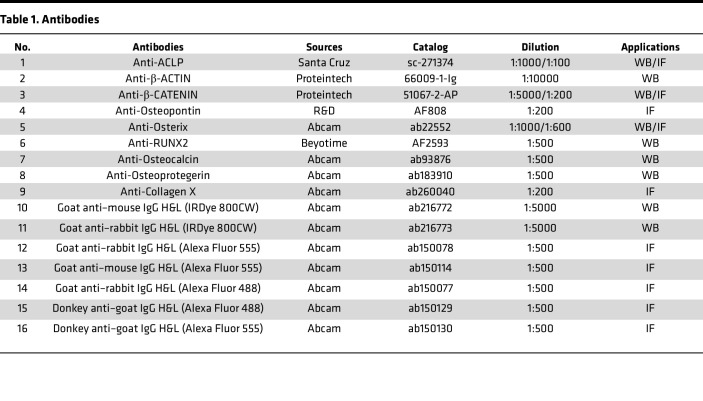
Antibodies

**Table 2:**
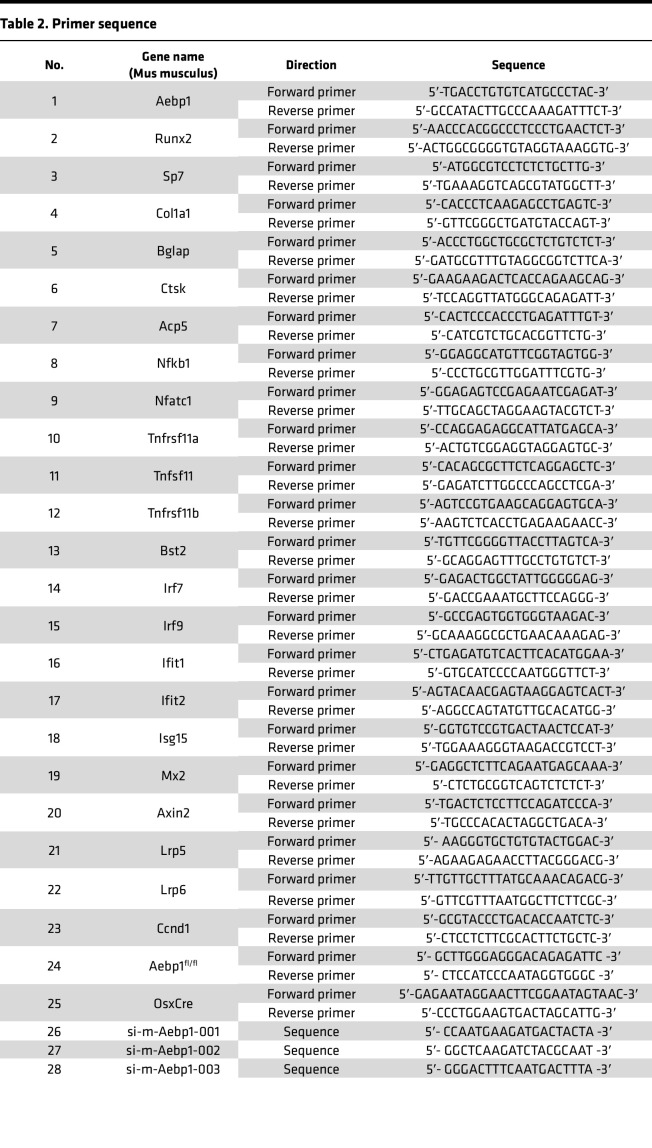
Primer sequence

## Version 1 11/13/2025

In-Press Preview

## Version 2 01/23/2026

Electronic publication
