# Supplementary material for: Aebp1 loss in osteoprogenitors leads to skeletal defects resembling Ehlers-Danlos Syndrome by diminishing Wnt/β-catenin signaling
Source: JCI Insight. 2025 Nov 13;11(2):e191606. doi: 10.1172/jci.insight.191606 (PMC12892912; doi:10.1172/jci.insight.191606)
Supplement: Supplemental data [file jciinsight-11-191606-s142.pdf]

Supplementary Figures

Supplementary Fig 1. Construct *Aebp1* conditional knockout mice. A. Schematic map of the conditional knockout mouse construction strategy. B. Representative gel image of PCR amplification validating the genotyping of conditional knockout mice. C. Relative expression of Aebp1 in P0 *Aebp1*$^{f/f}$ and *Aebp1*^{OsxCre} mice femur (n=3. Student's t-test. The data are shown as means ± SD). D. Western blotting analyses of P0 pups bone tissue with indicated genotypes.

Supplementary Fig 2. Knockdown *Aebp1* in osteoblasts inhibits osteogenic differentiation. A. Relative expression of *Aebp1* in mouse osteoblasts after siRNA induction were assessed by qRT-PCR. (n=3. one-way ANOVA. The data are shown as means ± SD) B. Relative expressions of *Sp7*, *Col1a1*, and *Bglap* in mouse osteoblasts after siRNA-induced osteogenic differentiation were assessed by qRT-PCR. (n=3. Student's t-test. The data are shown as means ± SD) C. Western blot analysis of ACLP, COL1A1, RUNX2, and OCN proteins levels in mouse osteoblasts after siRNA-induced osteogenic differentiation. D-E. Representative ALP (D) and ARS (E) staining of mouse osteoblasts after siRNA-induced osteogenic differentiation. Scale bar: 500 μm. F. Western blot analysis of ACLP, COL1A1 and OCN protein levels of mouse osteoblasts in *Aebp1*$^{f/f}$ and *Aebp1*^{OsxCre} mice. G. Western blot analysis of ACLP, COL1A1, OPG and OCN protein levels of *Aebp1*$^{f/f}$ mice osteoblasts treated with ad-GFP or ad-Cre. H. Representative ALP staining of *Aebp1*$^{f/f}$ mice osteoblasts treated with ad-GFP or ad-Cre.

Supplementary Fig 3. Osteocyte phenotype in *Aebp1*^{OsxCre} mice. A. Dendrite formation of osteocytes stained by phalloidin. Scale bar: 20 μ m. B. Quantification of dendrite numbers and dendrite length of the femur sections from 6-week-old mice with the indicated genotypes. (n=3. Student's t-test. The data are shown as means ± SD)

Supplementary Fig 4. Single-cell analysis of *Aebp1*$^{f/f}$ and *Aebp1*^{OsxCre} mice. A. Quality control plots of scRNA-seq data among each sample. B. Bubble plots of expression level of canonical osteoblast marker genes including *Alpl*, *Col1a1*, *Sp7*, *Bglap*, and *Ibsp* among each sample. C. Re-organized clusters of canonical osteoblastic genes expressed cells subset from femoral scRNA-seq data (a total of 5 clusters named S0-S4). D. Expression level of top 100 marker genes among each cluster shown in heatmap. E. Result of GO analysis with top 100 marker genes expressed in Cluster S1. F. Expression score of gene set of GO: Osteoblast differentiation in canonical osteoblastic genes expressed cells subset from femoral scRNA-seq data. G. Bubble plots of expression level of osteocyte marker genes including *Sost, Dmp1, Phex, Fam20c, Pdpn, Gja1* and *Dkk1* among each cluster. H. Results of GO analysis with top 100 fold change up-regulated DEGs in *Aebp1*$^{f/f}$ compare to *Aebp1*^{OsxCre} group in osteocytes. I. Distribution of chondrocyte marker genes-expressed cells shown in dot plots. J. Results of GO analysis with top 100 fold change up-regulated DEGs in *Aebp1*$^{f/f}$ compare to *Aebp1*^{OsxCre} group in chondrocytes.

Supplementary Fig 5. Pseudo-time analysis of *Aebp1*$^{f/f}$ and *Aebp1*^{OsxCre} mice. A. Differentiated trajectories of osteoblast cells. The direction of the arrow represents the direction of the pseudo-time. B. Distribution of the states of pseudo-time. The dots in each color represents each state, and each state is named numerically. C. Distribution of *Aebp1*$^{f/f}$ and *Aebp1*^{OsxCre} cells in pseudo-time. D. Dot

plots of expression level of *Alpl, Bglap, Col1a1, Runx2, Sox9* and *Sp7* among each state.

Supplementary Fig 6. Identification of pre-osteoclast cluster in sc-RNA-seq data. A. Bubble plots of expression level of *Tnfrsf11a* and *Csf1r* among each cluster. B. GO enrichment analysis results using top 100 markers of cluster 21 as input. C. GO enrichment analysis results of enriched upregulated markers in *Aebp1^{OsxCre}* group in cluster 21. D. Relative expression of Type I interferon signaling pathway related gene *Bst2, Irf7, Irf9, Ifit1, Ifit2, Isg15* and *Mx2* in 6-week-old mice femur were assessed by qRT-PCR. (n=4. Student's t-test. The data are shown as means ± SD)

Supplementary Fig 7. *Aebp1* deletion leads to reduction in Wnt/β-catenin signaling activity in the calvaria. A. Scatter plots shown data quality with different transform algorithms. B. Clustering of bulk-seq data of each calvaria sample.C. Principal component analysis (PCA) of the bulk RNA-seq data of *Aebp1$^{f/f}$* and *Aebp1^{OsxCre}* groups. (n=3 for each genotype.) D. Normalized counts of *Aebp1* in *Aebp1^{OsxCre}* groups compared to *Aebp1$^{f/f}$* groups. (n=3 for each genotype. Student's t-test. The data are shown as means ± SD) E. Volcano plot exhibits the DEGs of the *Aebp1^{OsxCre}* group compared to the *Aebp1$^{f/f}$* group. (n=3 for each genotype.) Blue dots show genes more highly expressed in the *Aebp1$^{f/f}$* group. Purple dots show genes more highly expressed in the *Aebp1^{OsxCre}* group. F. GO enrichment analysis of DEGs downregulated in the *Aebp1^{OsxCre}* group. G. Normalized counts of osteoblasts gene in *Aebp1^{OsxCre}* groups compared to *Aebp1$^{f/f}$* groups. (n=3 for each genotype. Student's t-test. The data are shown as means ± SD) H. Normalized counts of Wnt pathway gene in *Aebp1^{OsxCre}* groups compared to *Aebp1$^{f/f}$* groups. (n=3 for each genotype. Student's t-test. The data are shown as means ± SD)

Supplementary Fig 8. BIO treatment alleviate osteoblast and osteoclast abnormalities in vivo. A. Representative Masson's trichrome staining of femurs trabecular bone from 6-week-old mice with indicate genotype. Scale bar: 100 μm. B. Quantification of N.Ob/B.Pm of A. (n=3 for each genotype. one-way ANOVA. The data are shown as means ± SD). C. Representative Masson's trichrome staining of femurs cortical bone from 6-week-old mice with indicate genotype. Scale bar: 100 μm. D. Quantification of cortical bone thickness of C. (n=3 for each genotype. one-way ANOVA. The data are shown as means ± SD). E. Representative TRAP staining of femurs from 6-week-old mice. Scale bar: 100 μm. F-G. Quantitative analysis of TRAP staining of E. (n=3 for each genotype. one-way ANOVA. The data are shown as means ± SD).

Supplementary Fig 9. Osteoblast differentiation suppression of Aebp1 deletion was reversed by BIO treatment. A. Representative ARS staining of mouse osteoblasts after osteogenic differentiation with indicate genotype and treatment. B. Relative expressions of *Runx2, Sp7*, *Col1a1*, and *Bglap* in mouse osteoblasts after osteogenic differentiation were assessed by qRT-PCR. (n=3. one-way ANOVA. The data are shown as means ± SD)

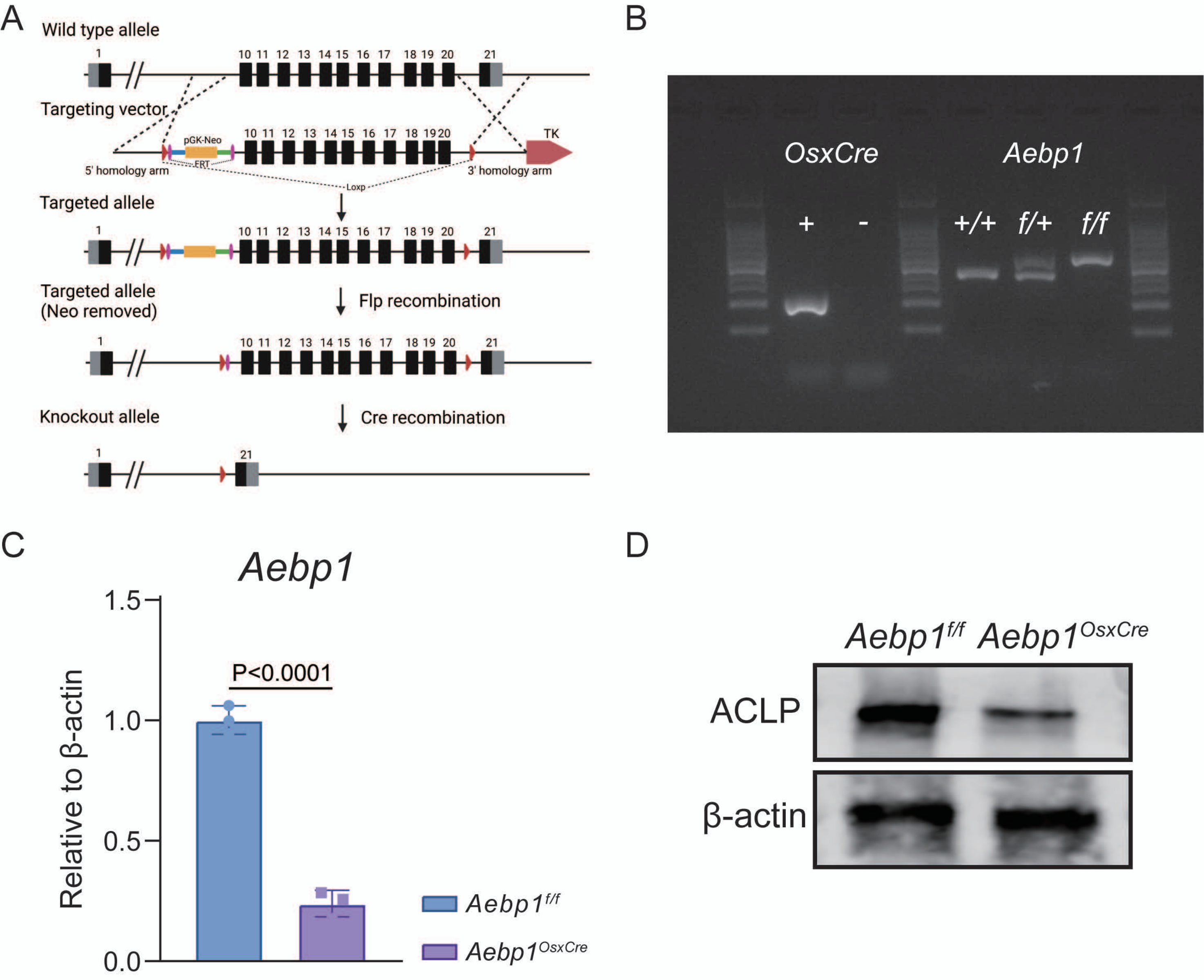
A
Wild type allele
Targeting vector
pGK-Neo
FRT
TK
5' homology arm
3' homology arm
Loxp
Targeted allele
Targeted allele
(Neo removed)
Flp recombination
Knockout allele
Cre recombination
B
OsxCre
Aebp1
+
-
+/+
f/+
f/f
C
Aebp1
P<0.0001
Relative to β-actin
1.5
1.0
0.5
0.0
Aebp1f/f
Aebp1OsxCre
D
Aebp1f/f
Aebp1OsxCre
ACLP
β-actin

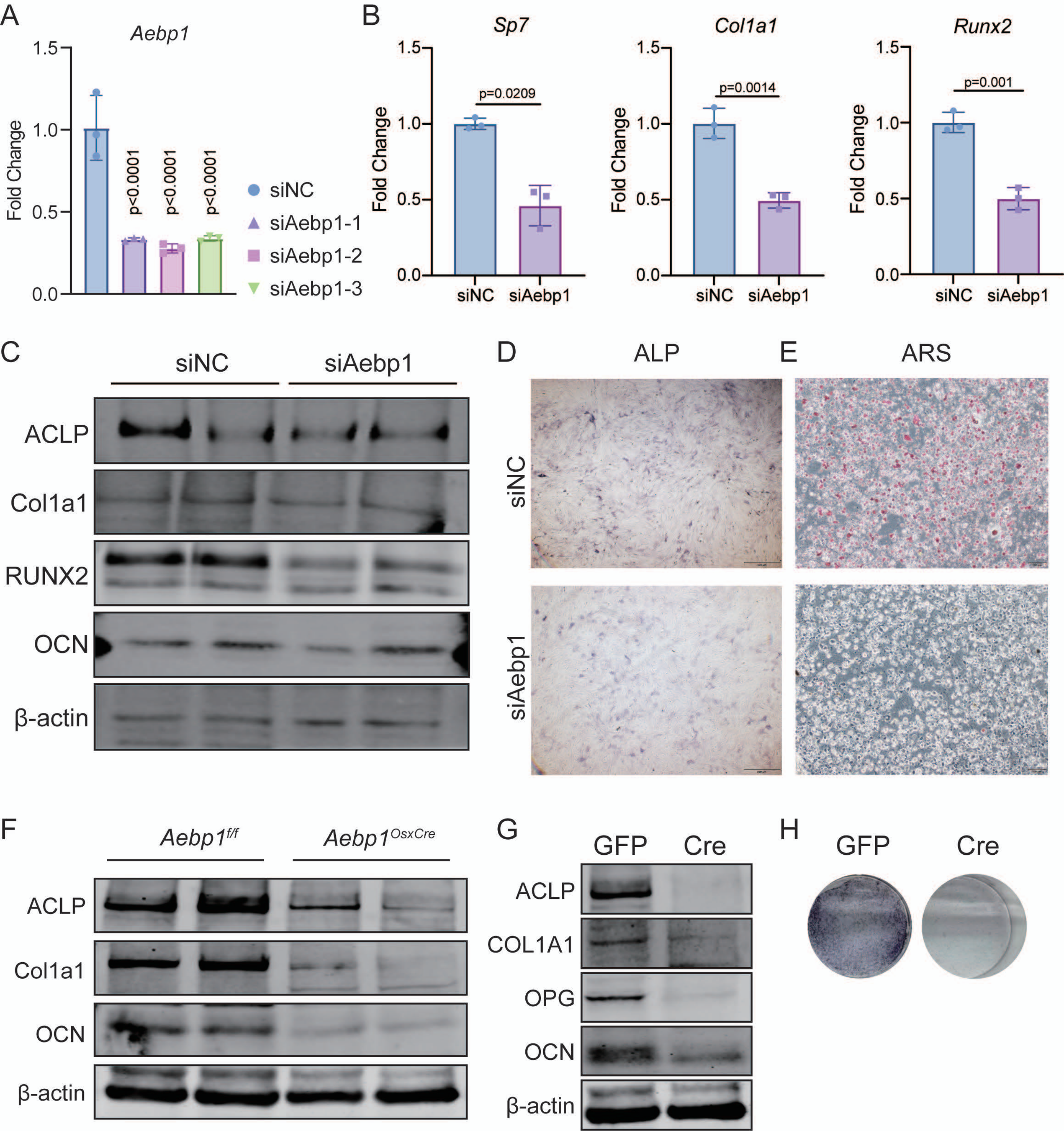

A
Aebp1
Fold Change
1.5
1.0
0.5
0.0
p<0.0001
p<0.0001
p<0.0001
siNC
siAebp1-1
siAebp1-2
siAebp1-3
B
Sp7
Col1a1
Runx2
Fold Change
p=0.0209
p=0.0014
p=0.001
siNC
siAebp1
C
siNC
siAebp1
ACLP
Col1a1
RUNX2
OCN
β-actin
D
ALP
E
ARS
siNC
siAebp1
F
Aebp1f/f
Aebp1OsxCre
ACLP
Col1a1
OCN
β-actin
G
GFP
Cre
ACLP
COL1A1
OPG
OCN
β-actin
H
GFP
Cre

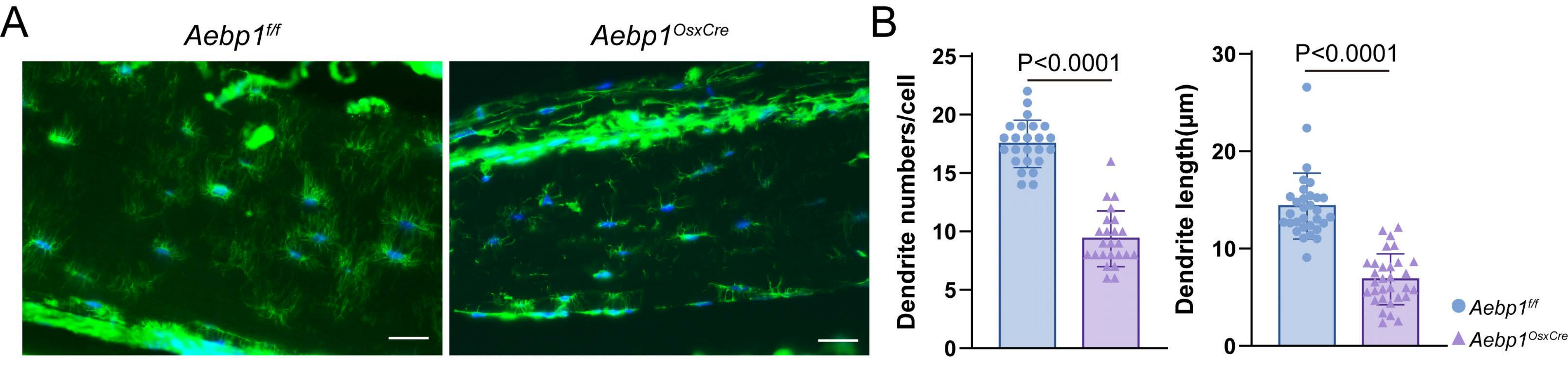
A
Aebp1f/f
Aebp1OsxCre
B
P<0.0001
Dendrite numbers/cell
25
20
15
10
5
0
P<0.0001
Dendrite length(μm)
30
20
10
0
Aebp1f/f
Aebp1OsxCre

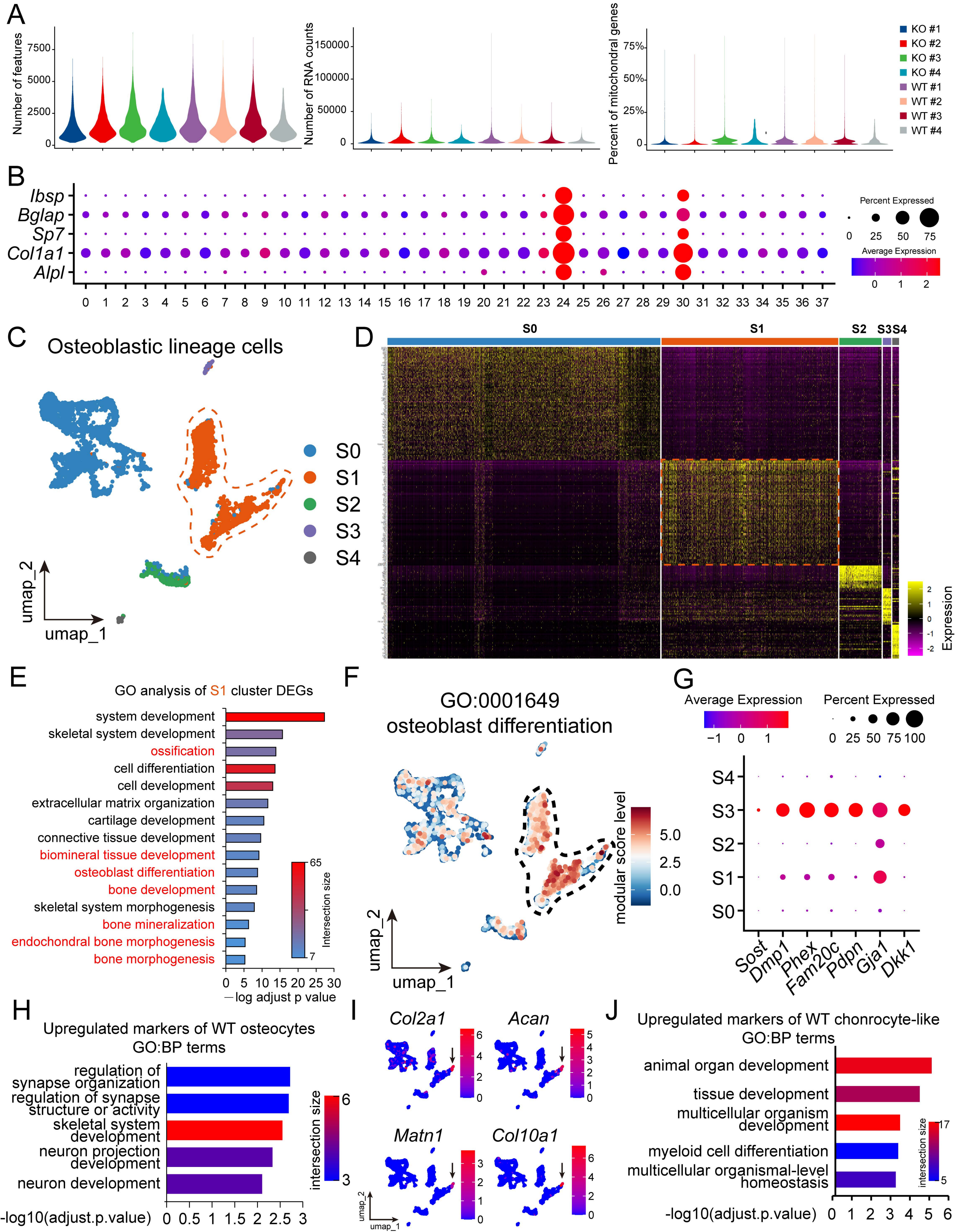

A
Number of features
Number of RNA counts
Percent of mitochondrial genes
KO #1
KO #2
KO #3
KO #4
WT #1
WT #2
WT #3
WT #4
B
Ibsp
Bglap
Sp7
Col1a1
Alpl
Percent Expressed
Average Expression
C
Osteoblastic lineage cells
S0
S1
S2
S3
S4
umap_2
umap_1
D
S0
S1
S2
S3
S4
Expression
E
GO analysis of S1 cluster DEGs
system development
skeletal system development
ossification
cell differentiation
cell development
extracellular matrix organization
cartilage development
connective tissue development
biomineral tissue development
osteoblast differentiation
bone development
skeletal system morphogenesis
bone mineralization
endochondral bone morphogenesis
bone morphogenesis
Intersection size
—log adjust p value
F
GO:0001649
osteoblast differentiation
modular score level
umap_2
umap_1
G
Average Expression
Percent Expressed
S4
S3
S2
S1
S0
Sost
Dmp1
Phex
Fam20c
Pdpn
Gja1
Dkk1
H
Upregulated markers of WT osteocytes
GO:BP terms
regulation of synapse organization
regulation of synapse structure or activity
skeletal system development
neuron projection development
neuron development
intersection size
-log10(adjust.p.value)
I
Col2a1
Acan
Matn1
Col10a1
umap_2
umap_1
J
Upregulated markers of WT chonrocyte-like
GO:BP terms
animal organ development
tissue development
multicellular organism development
myeloid cell differentiation
multicellular organismal-level homeostasis
intersection size
-log10(adjust.p.value)

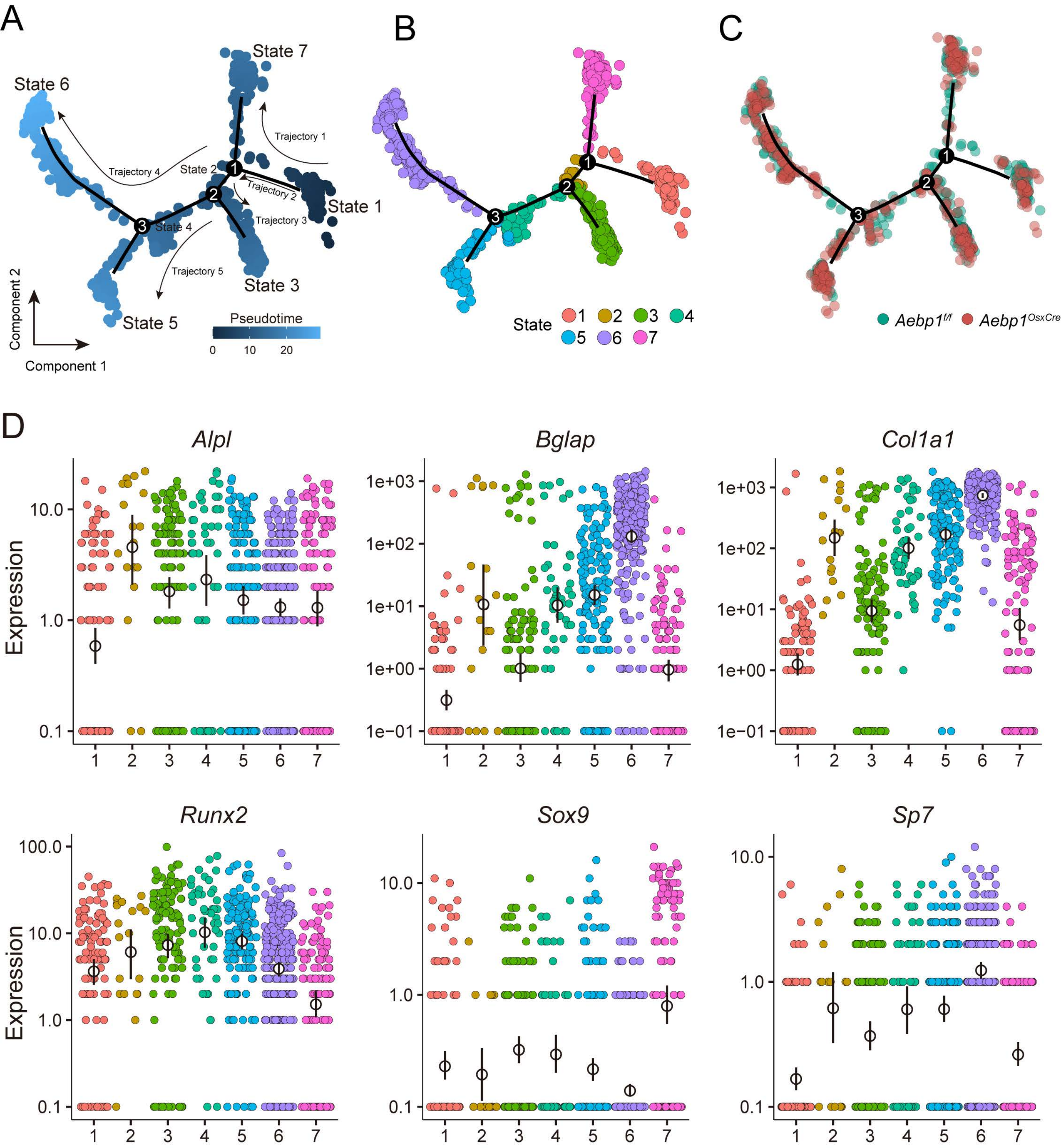
A
State 7
State 6
Trajectory 1
Trajectory 4
State 2
Trajectory 2
State 1
Trajectory 3
State 4
Trajectory 5
State 3
State 5
Component 2
Component 1
Pseudotime
0 10 20
B
State 1 2 3 4 5 6 7
C
Aebp1f/f Aebp1OsxCre
D
Alpl
Bglap
Col1a1
Runx2
Sox9
Sp7
Expression

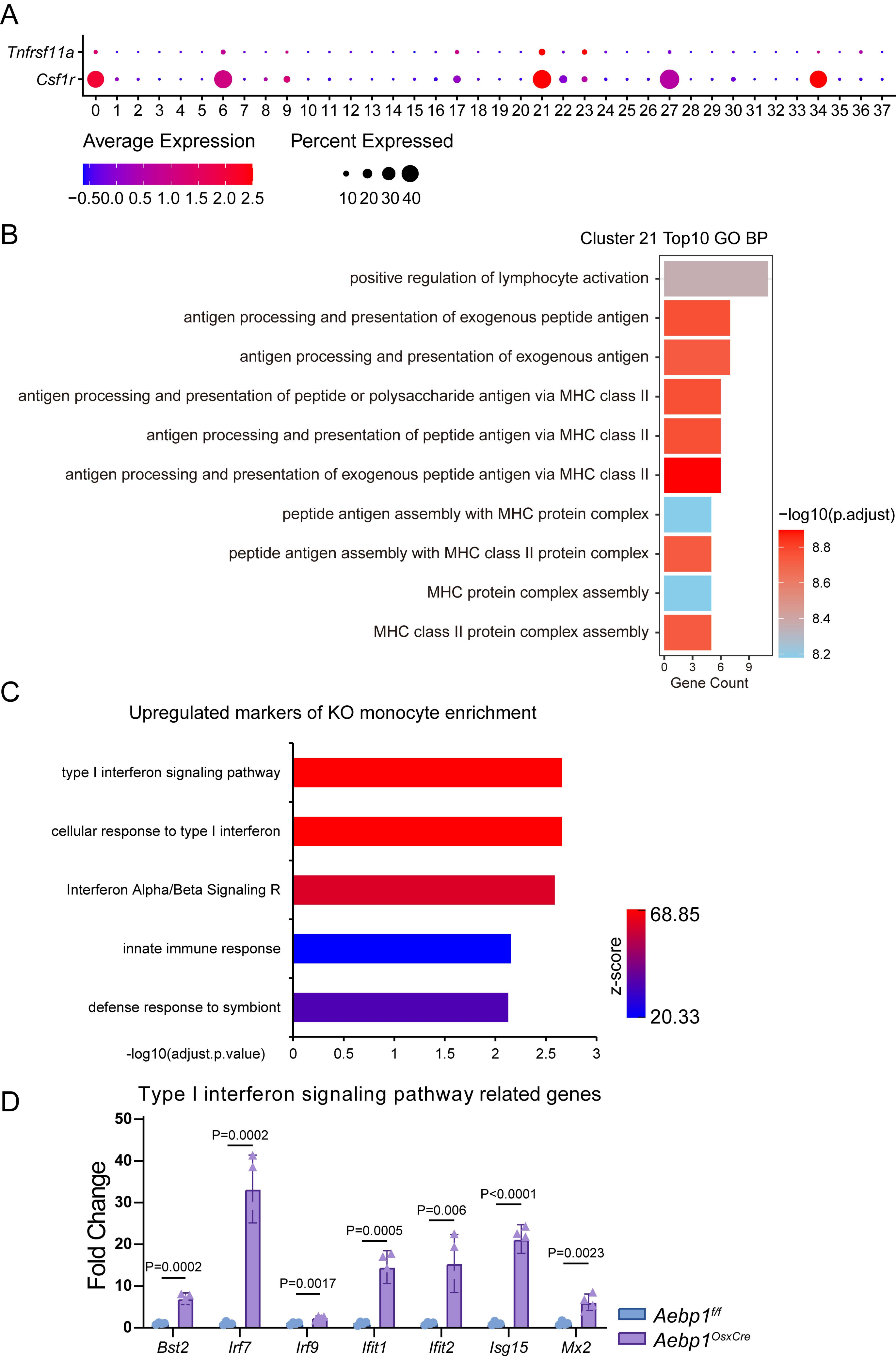
A
Tnfrsf11a
Csf1r
0 1 2 3 4 5 6 7 8 9 10 11 12 13 14 15 16 17 18 19 20 21 22 23 24 25 26 27 28 29 30 31 32 33 34 35 36 37
Average Expression
−0.50.0 0.5 1.0 1.5 2.0 2.5
Percent Expressed
10 20 30 40
B
Cluster 21 Top10 GO BP
positive regulation of lymphocyte activation
antigen processing and presentation of exogenous peptide antigen
antigen processing and presentation of exogenous antigen
antigen processing and presentation of peptide or polysaccharide antigen via MHC class II
antigen processing and presentation of peptide antigen via MHC class II
antigen processing and presentation of exogenous peptide antigen via MHC class II
peptide antigen assembly with MHC protein complex
peptide antigen assembly with MHC class II protein complex
MHC protein complex assembly
MHC class II protein complex assembly
0 3 6 9
Gene Count
−log10(p.adjust)
8.8
8.6
8.4
8.2
C
Upregulated markers of KO monocyte enrichment
type I interferon signaling pathway
cellular response to type I interferon
Interferon Alpha/Beta Signaling R
innate immune response
defense response to symbiont
-log10(adjust.p.value)
0 0.5 1 1.5 2 2.5 3
z-score
68.85
20.33
D
Type I interferon signaling pathway related genes
Fold Change
50
40
30
20
10
0
P=0.0002
P=0.0002
P=0.0017
P=0.0005
P=0.006
P<0.0001
P=0.0023
Bst2
Irf7
Irf9
Ifit1
Ifit2
Isg15
Mx2
Aebp1f/f
Aebp1OsxCre

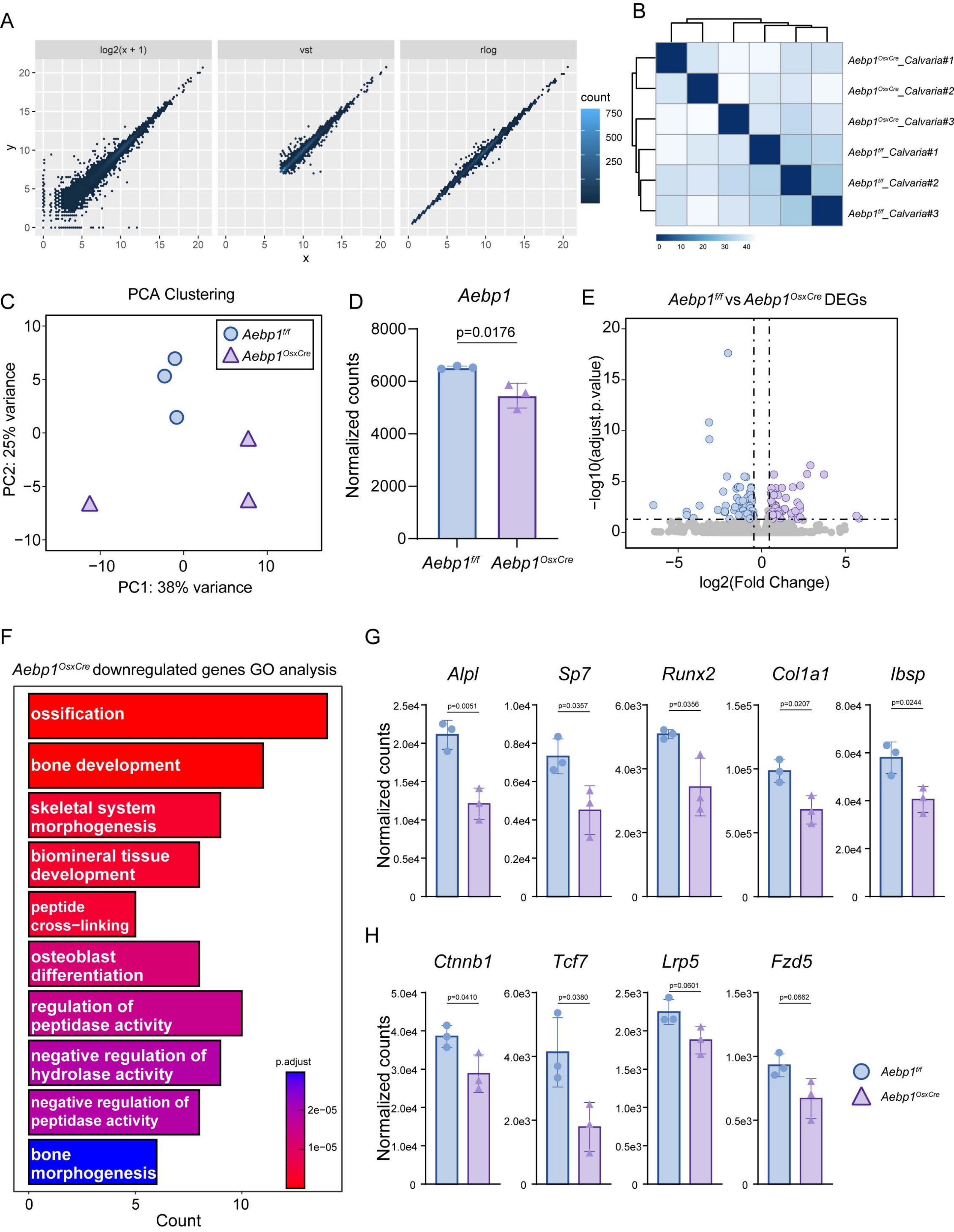
A
log2(x + 1)
vst
rlog
count
750
500
250
x
y
B
Aebp1^OsxCre_Calvaria#1
Aebp1^OsxCre_Calvaria#2
Aebp1^OsxCre_Calvaria#3
Aebp1^f/f_Calvaria#1
Aebp1^f/f_Calvaria#2
Aebp1^f/f_Calvaria#3
C
PCA Clustering
Aebp1^f/f
Aebp1^OsxCre
PC2: 25% variance
PC1: 38% variance
D
Aebp1
p=0.0176
Normalized counts
Aebp1^f/f
Aebp1^OsxCre
E
Aebp1^f/f vs Aebp1^OsxCre DEGs
-log10(adjust.p.value)
log2(Fold Change)
F
Aebp1^OsxCre downregulated genes GO analysis
ossification
bone development
skeletal system morphogenesis
biomineral tissue development
peptide cross−linking
osteoblast differentiation
regulation of peptidase activity
negative regulation of hydrolase activity
negative regulation of peptidase activity
bone morphogenesis
p.adjust
2e−05
1e−05
Count
G
Alpl
p=0.0051
Sp7
p=0.0357
Runx2
p=0.0356
Col1a1
p=0.0207
Ibsp
p=0.0244
Normalized counts
H
Ctnnb1
p=0.0410
Tcf7
p=0.0380
Lrp5
p=0.0601
Fzd5
p=0.0662
Normalized counts
Aebp1^f/f
Aebp1^OsxCre

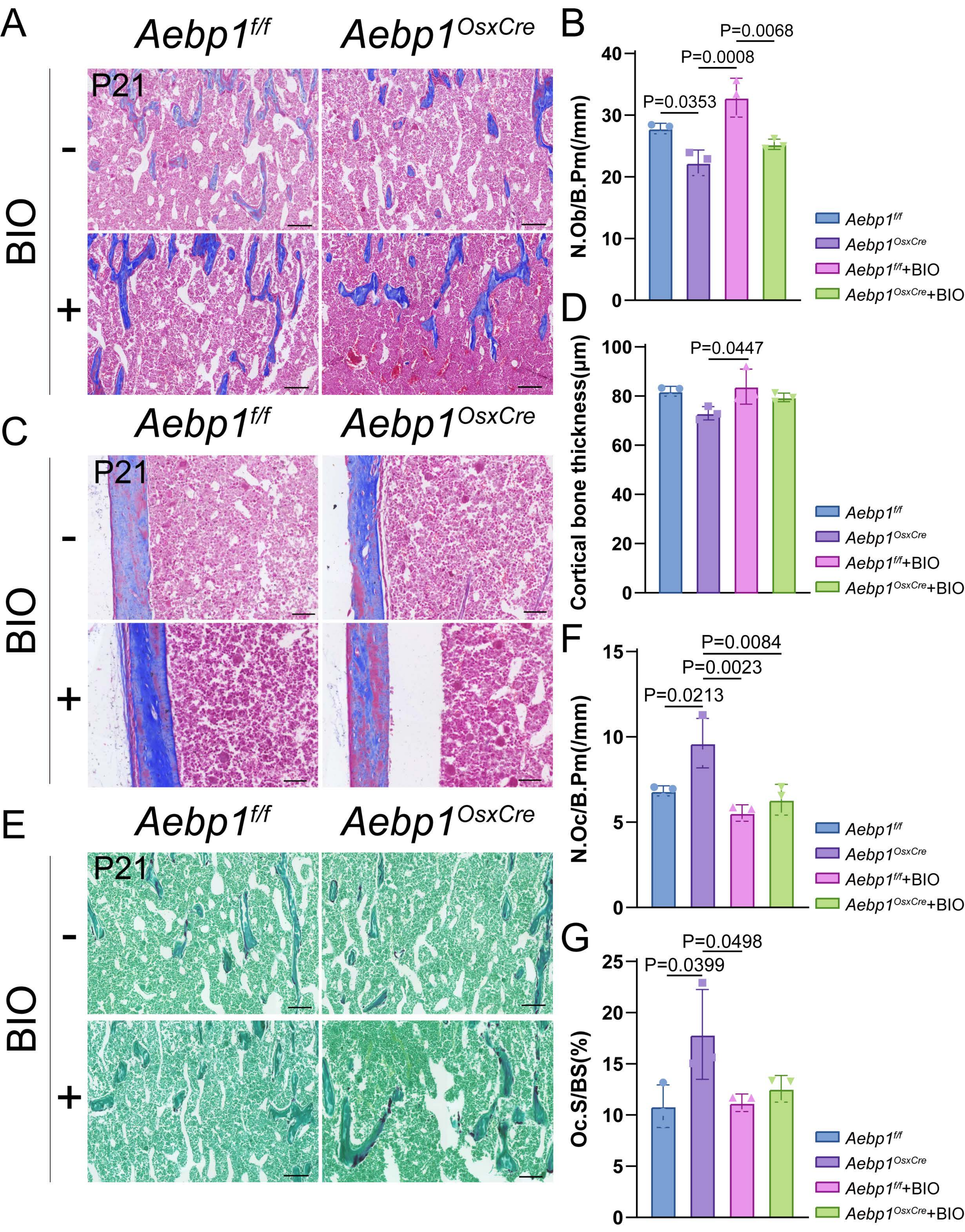

A
Aebp1f/f
Aebp1OsxCre
P21
BIO
-
+
B
N.Ob/B.Pm(/mm)
P=0.0353
P=0.0008
P=0.0068
Aebp1f/f
Aebp1OsxCre
Aebp1f/f+BIO
Aebp1OsxCre+BIO
C
D
Cortical bone thickness(μm)
P=0.0447
E
F
N.Oc/B.Pm(/mm)
P=0.0213
P=0.0023
P=0.0084
G
Oc.S/BS(%)
P=0.0399
P=0.0498

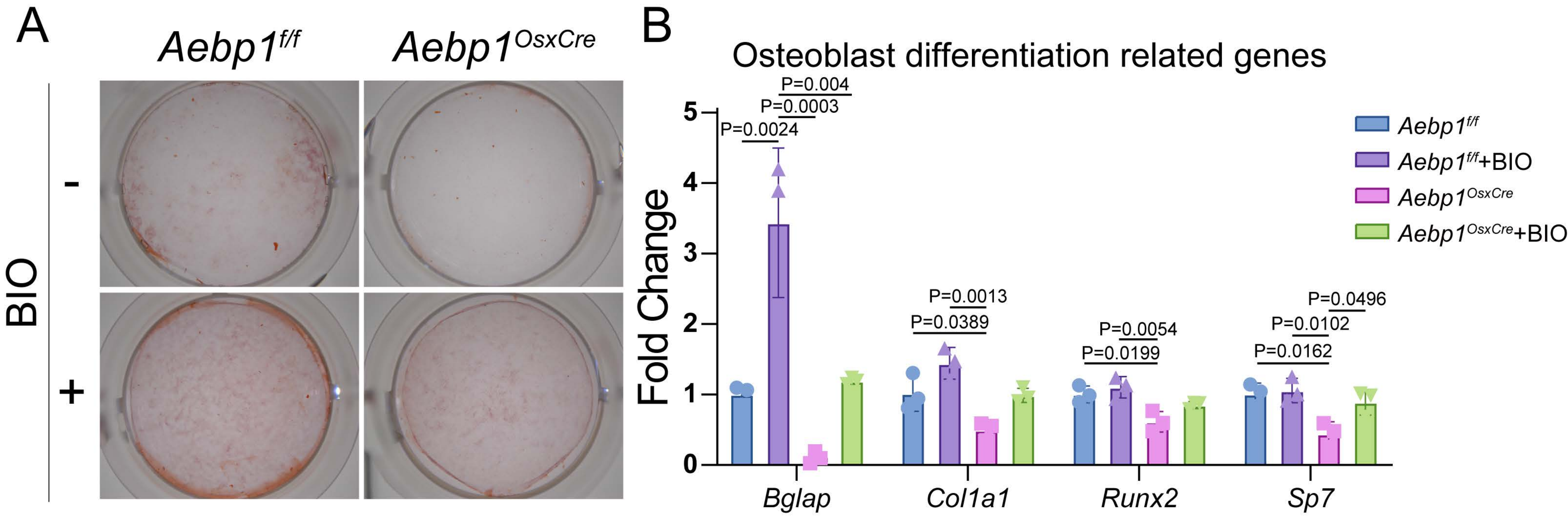
A
Aebp1f/f
Aebp1OsxCre
BIO
-
+
B
Osteoblast differentiation related genes
Fold Change
P=0.004
P=0.0003
P=0.0024
P=0.0013
P=0.0389
P=0.0054
P=0.0199
P=0.0496
P=0.0102
P=0.0162
Bglap
Col1a1
Runx2
Sp7
Aebp1f/f
Aebp1f/f+BIO
Aebp1OsxCre
Aebp1OsxCre+BIO
