# Supplementary material for: Aebp1 loss in osteoprogenitors leads to skeletal defects resembling Ehlers-Danlos Syndrome by diminishing Wnt/β-catenin signaling
Source: JCI Insight. 2025 Nov 13;11(2):e191606. doi: 10.1172/jci.insight.191606 (PMC12892912; doi:10.1172/jci.insight.191606)

Fig.1D

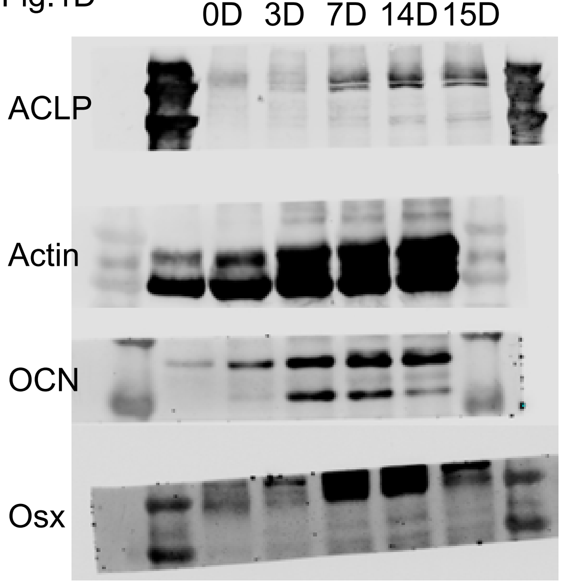

Fig.6K

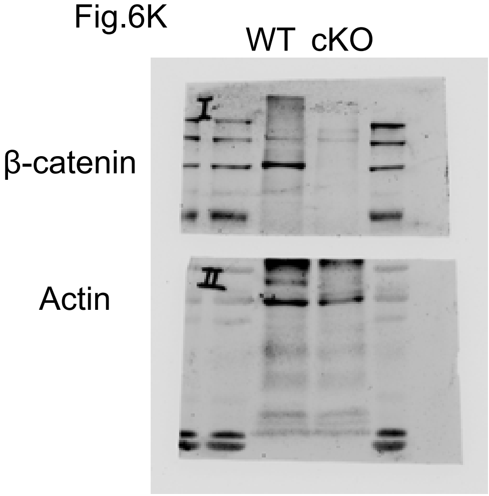

Fig.6N

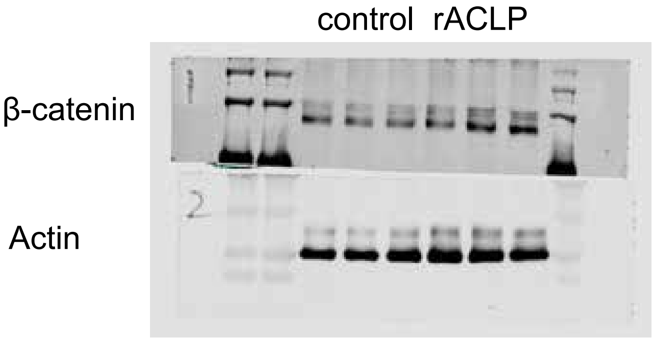

SF.1D

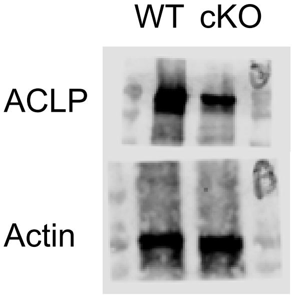

SF.2C

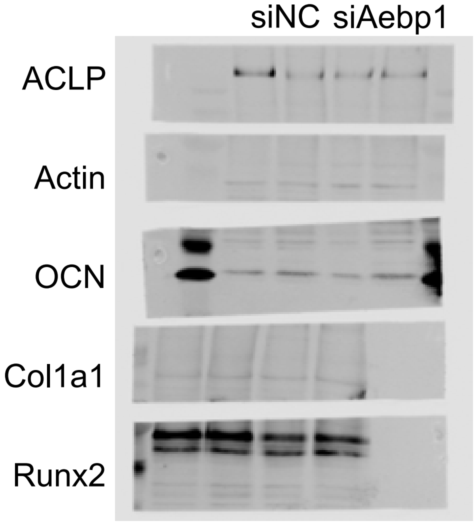

SF.2F

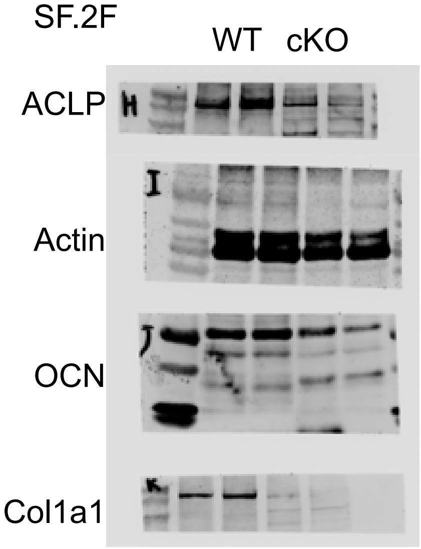

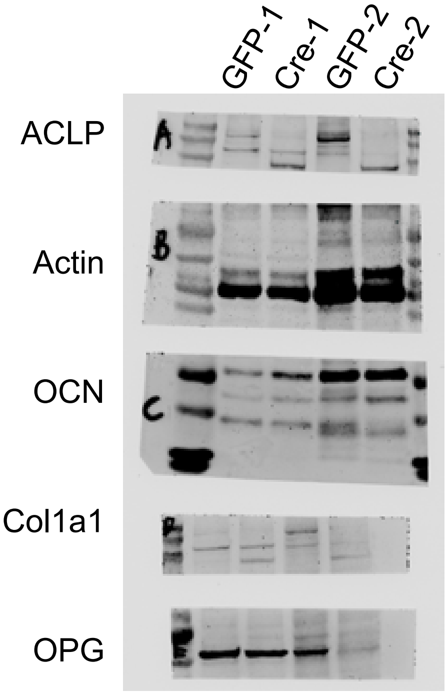

Supplement: Unedited blot and gel images [file jciinsight-11-191606-s143.pdf]
